# Generative Artificial Intelligence in the Metaverse Era: A Review on Models and Applications

**DOI:** 10.34133/research.0804

**Published:** 2025-08-19

**Authors:** Han Zhou, Xinyi Chen, Jin Li, Zichen Zhang, Yao Fu, Mailyn Pérez Liva, Dov Greenbaum, Pan Hui

**Affiliations:** ^1^ School of Instrumentation and Optoelectronic Engineering, Beihang University, Beijing 100191, China.; ^2^ National Key Lab of Spintronics, School of Integrated Circuit Science and Engineering, Beihang University, Beijing 100191, China.; ^3^ Shanxi Key Laboratory of Advanced Semiconductor Optoelectronic Devices and Integrated Systems, Shanxi 048000, China.; ^4^ The Changchun Institute of Optics, Fine Mechanics and Physics, Chinese Academy of Sciences, Changchun 130000, China.; ^5^ Department of Structure of Matter, Thermal Physics and Electronics, CEI Moncloa, Universidad Complutense de Madrid, 28040 Madrid, Spain.; ^6^ Department of Molecular Biophysics and Biochemistry, Yale University, New Haven, CT 06520, USA.; ^7^ The HKUST-DT System and Media Lab, Hong Kong University of Science and Technology, Hong Kong 999077, China.

## Abstract

The Metaverse is a decentralized, immersive 3-dimensional virtual environment that merges the physical and virtual worlds, fundamentally transforming digital interaction and garnering widespread attention. However, its primary platforms face challenges such as low-quality content and underdeveloped virtual environments, leading to a subpar user experience. Artificial intelligence-generated content (AIGC) has emerged as a key driver in Metaverse development, enabling the efficient and cost-effective creation of digital content. AIGC also promotes personalized content, further enhancing the appeal of the Metaverse. Although AIGC holds great promise, comprehensive investigations into its underlying models and applications remain limited. This study begins with the core neural network architectures of generative AI and examines the relationship between the Metaverse and AIGC. It delves into the deep learning technologies that support AIGC, providing both qualitative and quantitative analyses of their advantages, limitations, and hardware constraints. We also review existing practical applications of the Metaverse, highlighting the challenges and future opportunities in key domains such as healthcare and education. The research concludes that while AIGC can markedly accelerate the development of the Metaverse, its technology must be more closely aligned with development needs to deliver a truly immersive experience. The integration of AIGC and the Metaverse represents the convergence of artificial intelligence, computer graphics, and human–computer interaction. This interdisciplinary synergy has the potential to redefine the way we create, interact with, and experience digital environments, pushing the boundaries of creativity and immersion.

## Introduction

The Metaverse is envisioned as a persistent, immersive, and interconnected virtual environment that allows users to interact in real time with a strong sense of presence and agency [1–4]. Since the concept was formally introduced in the Metaverse Roadmap (2007) [5,6] and later brought into the spotlight by Meta in 2021, it has attracted widespread attention across academia, industry, and society. By integrating cutting-edge technologies such as virtual reality (VR), augmented reality (AR), haptic feedback, and real-time rendering, the Metaverse is poised to reshape diverse fields, including education [7], healthcare [8], e-commerce, and entertainment [9].

However, building such complex, dynamic virtual worlds pose a fundamental challenge: the creation of massive amounts of diverse and high-quality digital content—ranging from 3-dimensional (3D) environments and digital humans to interactive scenarios and adaptive narratives [10–12]. Traditional content creation methods are labor-intensive and time-consuming and require significant domain expertise, making them inadequate for the real-time and large-scale demands of the Metaverse [13–17].

**Fig. 1.**
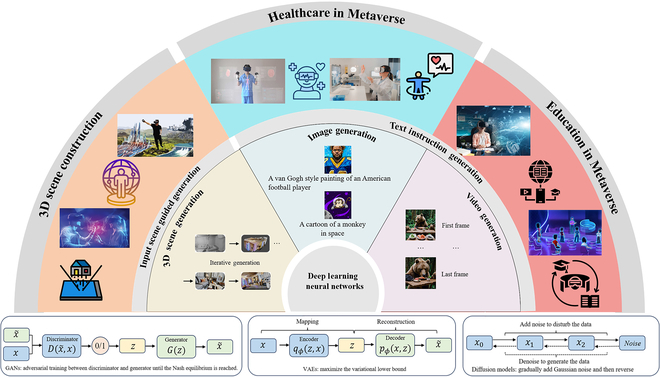
The application of AIGC driven by deep learning models, and its use in the Metaverse. The relationship between AIGC and the Metaverse. AIGC facilitates the construction of the Metaverse, while the demands of the Metaverse drive the development of AIGC.

In this context, generative artificial intelligence (GAI)—particularly artificial intelligence-generated content (AIGC)—has emerged as a transformative solution. AIGC has demonstrated significant impact across diverse fields such as computer vision [18–22], natural language processing (NLP) [23–25], music composition [18,26,27], and medicine discovery [28–30]—validating its capacity to synthesize complex, realistic, and semantically meaningful data. In NLP, text generation models have demonstrated their utility in applications ranging from conversational systems to content creation. For example, the OpenAI API provides access to models such as GPT-4, which are suitable for text generation, summarization, dialogue systems, and more. As shown in Fig. 1, these capabilities not only alleviate the content scarcity problem but also lay the foundation for dynamic and immersive virtual experiences. In the context of the Metaverse, these capabilities enable the creation of highly realistic and adaptive virtual environments, making GAI a key driver in shaping the future of immersive digital worlds.

Yet, despite this momentum, deploying AIGC in Metaverse applications presents substantial challenges. Key issues include data quality control, model interpretability, computational efficiency, ethical governance, and cross-domain integration. Moreover, there is a lack of standardized evaluation frameworks to assess the fidelity, immersion, and utility of AIGC-generated content in large-scale interactive environments.

Against this backdrop, the necessity of this review is twofold. First, there is an urgent need to systematically organize the fast-growing body of research at the intersection of AIGC and the Metaverse. This includes understanding foundational model architectures, surveying current applications, and identifying promising generation pipelines. Second, it is critical to explore the existing limitations and research gaps that hinder the full potential of AIGC in shaping future immersive ecosystems. It is worth noting that, to provide a comprehensive technical background, we selectively cite existing review articles that focus on different aspects of generative models, application domains, and theoretical foundations. Each cited review offers unique insights—ranging from fundamental model designs to application-specific implementations—which collectively support the discussions and analyses presented in this paper.

By outlining the current advantages and gaps in the application of AIGC in the Metaverse, we advocate for the development of more robust and nuanced evaluation frameworks. These frameworks should not only address the complexity of virtual environments but also assess the quality of AIGC-generated content, the immersiveness of user experiences, and the innovative potential of future model-centric advancements. This research aims to establish a foundational knowledge base for academics and industry practitioners, thereby promoting future progress in evaluation methods and application technologies of AIGC within the metaverse.

The main contributions of our paper are threefold:

1. It provides an overviews of key GAI models and technological advancements. We provide a comprehensive survey of foundational and emerging GAI models, including generative adversarial networks (GANs), variational autoencoders (VAEs), Diffusion models, Transformers, and Mamba. By analyzing their architectural designs, strengths, and limitations, we explain how each model contributes to content generation for the Metaverse. We also highlight recent hybrid modeling approaches that combine the advantages of multiple architectures to enhance generative performance and efficiency. These insights offer a solid technical foundation for researchers and developers interested in applying generative models to immersive virtual environments.
2. It explores successful AIGC applications and their specific roles in shaping the Metaverse. We systematically review the practical applications of AIGC in shaping various components of the Metaverse. This includes virtual scene construction, avatar creation, interactive dialogue systems, and real-time video or 3D generation. By analyzing successful cases in domains such as healthcare, education, entertainment, and digital fashion, we reveal how AIGC accelerates production pipelines, improves user experience, and enables personalized content creation. This paper bridges the gap between model-level understanding and application-level deployment, demonstrating the real-world viability of GAI.
3. It identifies the challenges facing AIGC in the context of Metaverse development and offers future research directions for optimizing these technologies in immersive digital worlds. We identify current technical and practical challenges that hinder the full-scale adoption of AIGC in the Metaverse, such as model interpretability, real-time inference constraints, evaluation metrics, ethical risks, and sustainability concerns. We further propose future research directions to address these issues, including lightweight generative model design, hybrid edge–cloud deployment, cross-modal evaluation frameworks, and bias mitigation strategies. These suggestions aim to support the responsible development of AIGC for immersive, scalable, and ethical Metaverse ecosystems.

## Materials and Methods

### GAI models and their connection to the Metaverse

In the development of the Metaverse, the creation and display of content plays a critical role. The immersive experience and appeal of the Metaverse depend largely on the variety and complexity of its digital content, which includes virtual environments, objects, and characters. AIGC helps overcome the issue of limited content within the Metaverse by providing an abundant if not potentially endless supply of digital assets to build virtual worlds, characters, objects, and events. As a result, AIGC is instrumental in accelerating the replication of the physical world in the Metaverse, enabling the production of boundless content and fostering organic, self-sustaining growth.

This section will organize an exploration of the most widely used generative deep learning frameworks in GAI, such as GANs, VAEs, and diffusion models, as well as cutting-edge foundational neural network architectures, including Transformer and Mamba. Notably, while examining these deep learning technologies, this paper will also highlight their connections to the Metaverse.

#### GANs and VAEs

GANs and VAEs are 2 fundamental generative models widely adopted in the AIGC field, which can serve as core components for virtual content and environment generation in the Metaverse [31]. GANs consist of 2 neural networks—a generator and a discriminator—that are trained simultaneously in a competitive setting. The generator maps random noise vectors to data-like outputs, aiming to synthesize realistic samples, while the discriminator attempts to distinguish between real data and synthetic samples. During training, both networks are optimized in a minimax game: the generator tries to fool the discriminator, while the discriminator learns to become more accurate. This adversarial process drives the generator to produce outputs that are increasingly indistinguishable from real data [32]. Figures 1 (lower left) and 2A illustrate this fundamental mechanism.

**Fig. 2.**
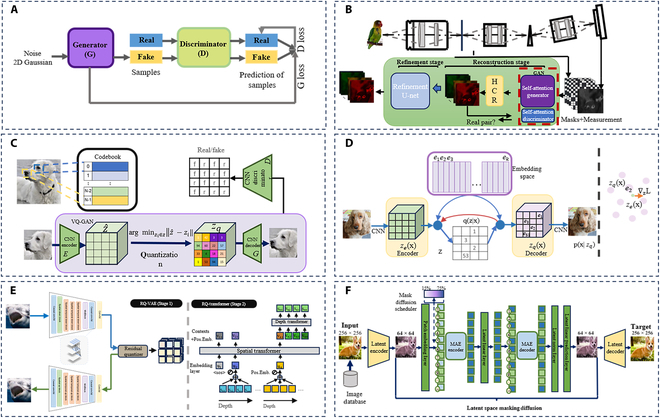
Advances in GANs and VAEs. Zoom in for a better view. (A) Basic GANs architecture [48]. (B) Spectral compressive imaging reconstruction with self-attention GAN [43]. (C) Convolutional VQGAN and autoregressive Transformer for high-resolution image synthesis [46]. (D) VQ-VAE: embedding space mapping and encoder gradient dynamics [47]. (E) An overall framework of RQ-transformer for text to images [200]. (F) The framework of latent space masking diffusion [49].

Despite their power, GANs often suffer from training instability and mode collapse, where the generator produces limited diversity [33,34]. To mitigate this, Wasserstein GAN (WGAN) replaced the original Jensen–Shannon divergence with the Wasserstein distance, improving convergence and gradient behavior [35,36].

To further improve the quality, diversity, and controllability of generated content, many advanced GAN variants have been developed. Deep Convolutional Generative Adversarial Networks (DCGANs) introduced convolutional layers into both the generator and discriminator to better exploit spatial information and stabilize training [37]. Conditional GAN (CGAN) incorporated label or attribute information as conditional inputs, enabling targeted generation of specific categories or styles [38]. StyleGAN introduced a style-based generator architecture that allows multi-level control over semantic attributes such as facial features or textures, enabling high-quality and editable image synthesis [39]. BigGAN scaled up the model and training dataset to achieve state-of-the-art performance in high-resolution and class-conditional image generation [40]. For multimodal and semantic-consistent generation, SPatially-Adaptive (DE)normalization and semantic region-adaptive normalization incorporated spatially adaptive normalization and semantic attention mechanisms, respectively, allowing detailed control over output layout and content [41,42].

In the field of scientific imaging, λ-Net [43] was proposed to generate hyperspectral images by learning the spectral–spatial correlation across multiple bands, enabling end-to-end synthesis of high-resolution spectral data. These advanced variants markedly expand the applicability of GANs in content creation, from photorealistic avatars to domain-specific reconstructions, and play a critical role in constructing immersive environments in the Metaverse.

VAEs, introduced by Kingma and Welling [44], represent one of the earliest techniques in unsupervised learning and generative modeling. The core framework of VAEs, as illustrated in the middle-lower panel of Fig. 1, merges probabilistic methods with the foundational structure of autoencoders. Unlike traditional autoencoders that solely compress data into a latent space and reconstruct it, VAEs further impose a prior distribution—typically a standard Gaussian—on the latent variables. This design enables the generation of new, diverse samples from the learned latent distribution. The architecture of VAEs consists of 2 main components: an encoder and a decoder. The encoder maps input data to a low-dimensional latent space, while the decoder reconstructs the original data from the latent representation. By enforcing a standard Gaussian prior in the latent space, VAEs promote regularity and smoothness in the generative process, aligning the outputs with the statistical properties of the input distribution. As a result, VAEs are capable of capturing meaningful data structures and generating novel samples that are consistent with the training data.

Early advancements in autoencoder architectures include denoising autoencoders [45], which enhance robustness by learning to reconstruct clean data from corrupted inputs, and convolutional autoencoders [39], which incorporate convolutional neural networks (CNNs) to address challenges in image data processing. Currently, VAE is often combined with other architectures, i.e., GAN, diffusion, and Transformer. Latent diffusion models (LDMs) have emerged as a powerful generative framework by integrating VAEs with diffusion models. The core idea behind LDMs is to shift the computationally expensive denoising and generation process from the high-dimensional pixel space to a compressed latent space, substantially improving efficiency without sacrificing generation quality. In this framework, a VAE is first trained to encode images into a compact latent representation that retains essential semantic and perceptual information. Once trained, the diffusion process operates within this latent space, where noise is gradually removed to generate coherent outputs. This design addresses the inefficiency of conventional diffusion models, which require many iterations in pixel space, and also reduces the memory footprint during training and inference. Figure 2 shows LDM variants used for image/video generation, including Vector Quantized Generative Adversarial Network (VQGAN) [46] and Vector Quantized-Variational AutoEncoder (VQVAE) [47]. A trained VAE can serve as a general compression model, with its latent space used to train multiple generative models and applied to other downstream tasks, enhancing feature representation capabilities. In Fig. 2B to F, we summarize other GAN- and VAE-based architectures for text/image/video generation [40,43,46–49].

Overall, these properties of GAN and VAE models enable them to generate realistic virtual characters, landscapes, environments, and objects in the Metaverse, greatly enriching the virtual experience.

#### Diffusion models

As introduced by Salimans et al. [41], diffusion models were initially proposed to improve the performance of GANs. Diffusion models are powerful tools for generating high-quality samples, through continuous development, and have become a star architecture in the AIGC field, showcasing the latest advancements in computer vision. The generator model of GAN must undergo a step from pure noise to a mirrored image (X_T_ → X₀), which is a source of instability during training.

Unlike GANs, as depicted in the bottom-right section of Fig. 1, the diffusion process is divided into 2 key stages: the forward process (diffusion) and the reverse process (denoising). During the forward process, data are progressively corrupted into a noisy state, while the reverse process focuses on reconstructing the original data from this noise. Training a diffusion model revolves around reducing the discrepancy between the generated outputs and real data samples. This is achieved by simulating a step-by-step transformation of random noise into structured, meaningful data. The model learns to reverse the diffusion process, effectively reconstructing coherent patterns from noise. By iteratively refining this process, the model becomes adept at generating high-quality, realistic samples that closely resemble the original data distribution. This approach not only ensures fidelity to real-world data but also enables the creation of diverse and novel outputs. Additionally, the iterative nature of diffusion models makes both their training and generation processes more stable.

**Fig. 3.**
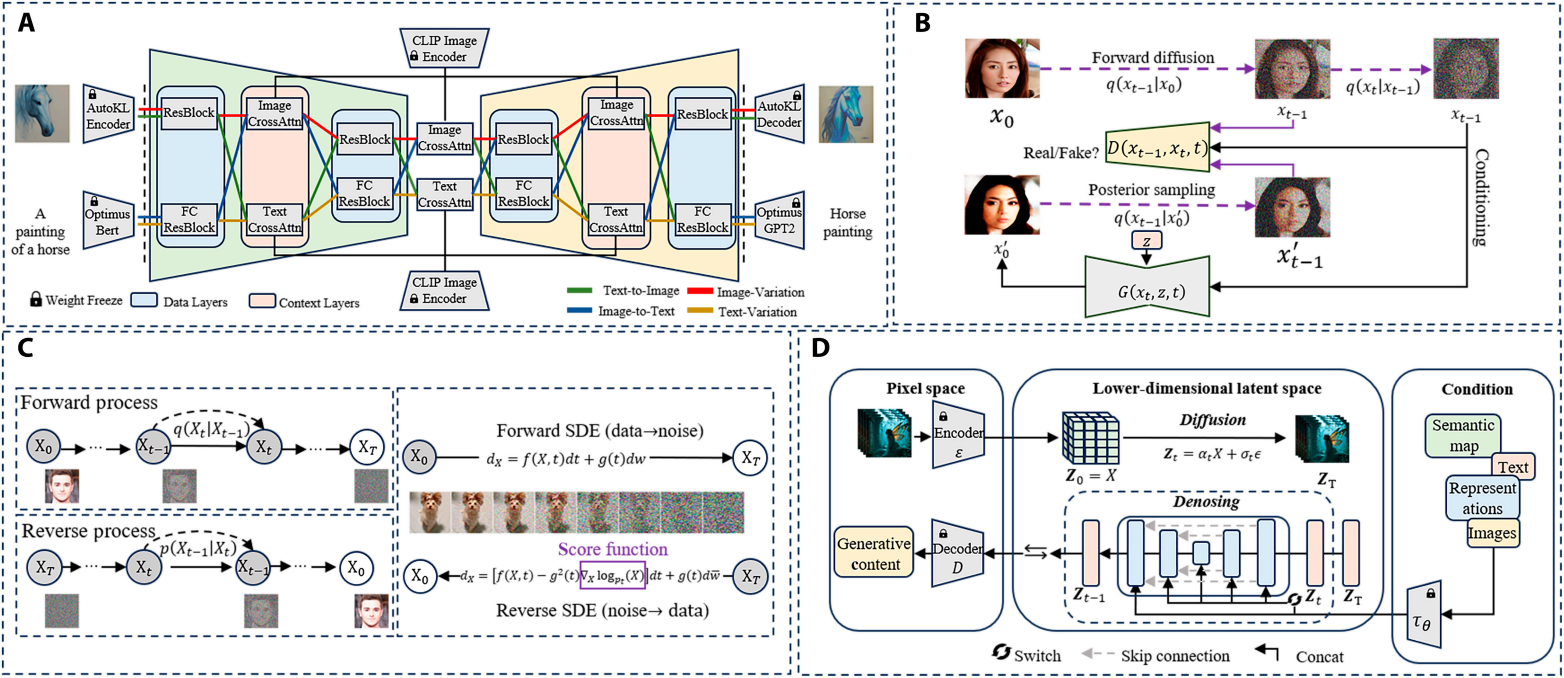
Advances of diffusion models. Zoom in for a better view. (A) Four-flow versatile diffusion (VD) framework for multi-task support [51]. (B) The overall framework of denoising diffusion GAN [53]. (C) The overall framework of DDPM [42] and score-based diffusion model via reverse-time stochastic differential equation (SDE) [50]. (D) Conditional LDMs with concatenation and cross-attention [52].

Diffusion models can be categorized into 3 main types, with several classic examples and their variants illustrated in Fig. 3 [42,50–53]. As shown in Fig. 3C, a denoising diffusion probabilistic model (DDPM) [42] utilizes 2 Markov chains to gradually corrupt data using Gaussian noise and learn to reverse this forward diffusion process by estimating the Markov transition kernel. Score-based generative models (SGMs), on the other hand, focus on the logarithmic density gradient of the data, referred to as the score function. Noise conditional score networks [54] introduce multi-scale noise perturbations and employ a neural network to estimate the score function across all noise levels simultaneously. This decoupling of training and inference steps enables flexible sampling. Score-based stochastic differential equations [50] generalize these concepts to a continuous framework, where noise perturbation and denoising are modeled as solutions to stochastic differential equations. This approach also demonstrates that probability flow ordinary differential equations can effectively describe the reverse process, further expanding the versatility of diffusion models.

Diffusion models have also found success in NLP tasks, where they can generate coherent and contextually relevant text sequences. By tokenizing sentences and converting them into word embeddings, these models leverage the diffusion process to learn and produce natural language outputs. They excel in handling a variety of complex NLP applications, including machine translation, question answering, search query completion, sentiment analysis, and text continuation. A prominent example is Diffusion-LM (language model) [55], which adapts diffusion models to the discrete nature of text data. This model addresses the challenge of applying continuous diffusion processes to discrete text by enabling fine-grained and controllable language generation. Experimental results demonstrate that Diffusion-LM achieves state-of-the-art performance across 6 controllable text generation tasks, showcasing its versatility and effectiveness in NLP applications. Diffusion models have demonstrated remarkable capabilities in generating images, videos, and even 3D objects from textual descriptions, showcasing their versatility in multimodal tasks. Prominent examples of text-to-image diffusion models include DALLE-2 [56], Imagen [57], versatile diffusion (VD) [51], and the widely accessible stable diffusion [51,52], which have set new benchmarks in generating high-quality visuals from text prompts. Beyond static images, diffusion models have been extended to video generation, as seen in Meta AI’s Make-A-Video [58] and ControlNet Video [59], which transform text inputs into dynamic video sequences.

In the realm of 3D content creation, diffusion models have been adapted to generate 3D objects from text descriptions, utilizing various representations such as point clouds, meshes, and neural radiance fields (NeRFs) [60]. For instance, DiffRF [61] introduces a diffusion model specifically designed for generating 3D radiance fields from text, while 3DFuse [62] focuses on creating 3D point clouds from 2D images. These advancements highlight the flexibility of diffusion models in handling diverse data formats and their potential to revolutionize fields like computer graphics, VR, and digital content creation. By bridging the gap between textual descriptions and complex visual outputs, diffusion models continue to push the boundaries of GAI, enabling more intuitive and creative applications.

Recently, there have been new developments in diffusion models; some generative architectures have emerged that attempt to combine the advantages of diffusion models and other generative models, i.e., creating a hybrid modeling approach. The goal of hybrid modeling is to integrate diffusion models with other generative models, aiming to enhance expressive power, improve sampling efficiency, or address specific limitations. DiffuseVAE [63] combines the strengths of standard VAEs and DDPMs by incorporating blurry images generated by the VAE into the diffusion sampling process. This integration allows for more efficient and high-quality image generation, leveraging the VAE’s ability to capture latent representations and the diffusion model’s capacity for fine-grained detail refinement. Latent score-based generative model (LSGM) [64] trains SGMs in the latent space of a VAE. By operating in a lower-dimensional latent space, LSGM generalizes SGMs to non-continuous data and enables smoother and more efficient learning. This approach not only reduces computational complexity but also improves the model’s ability to generate diverse and high-quality outputs.

Denoising diffusion GANs (DDGANs) [53] integrate conditional GANs into the DDPM framework, enabling the use of richer, multimodal distributions to guide the denoising process. This allows for larger steps in the denoising phase, markedly speeding up generation. On the other hand, DiffFlow [65] introduces flow-based techniques into the trajectories of PDE-driven diffusion models, making the forward steps adaptable through training. The inherent randomness from noise perturbation amplifies the expressive potential of normalized flows, while the trainable forward process shortens the overall diffusion path. This results in more efficient sampling and the ability to model distributions with sharper, more precise boundaries. DDGANs [53] diverge from traditional diffusion models by replacing the Gaussian assumption with a multimodal conditional distribution, pioneering the use of a GAN-based training objective in diffusion frameworks. This approach inherits the rapid sampling benefits of GANs. A key insight is that smaller denoising steps tend to produce Gaussian-like outputs, while larger steps lead to multimodal (peaked) distributions. To optimize sampling speed, DDGANs leverage multimodal distributions instead of relying on single-peaked Gaussians. Unlike adversarial distillation methods, which focus on distinguishing synthetic from real images, DDGANs utilize denoised “real” latent samples. However, the scalability limitations of GANs restrict DDGANs from being applied to large-scale datasets.

These hybrid architectures represent a significant step forward in generative modeling, as they combine the complementary strengths of different frameworks. By merging the stability and controllability of diffusion models with the efficiency and compact representations of VAEs or the adversarial training of GANs, these models open up new possibilities for faster sampling, improved performance, and broader applicability across various domains, such as image synthesis, video generation, and 3D content creation.

In addition to the methods mentioned above to improve diffusion computational efficiency, there are also strategies including fractional distillation sampling (SDS) to enhance fidelity, model pruning, knowledge distillation, and mixed training with GAN loss to stabilize learning and improve convergence speed. These advancements make diffusion models more suitable for real-time and interactive Metaverse scenarios, such as dynamic avatar generation and virtual scene synthesis.

In the Metaverse, diffusion models extend their capabilities beyond text, image, and video generation to crafting detailed virtual objects and immersive environments. They excel at simulating realistic natural phenomena, such as flowing water, smoke [66], intricate textures, and fine details of virtual assets [67]. Moreover, diffusion models can generate lifelike actions and behaviors for virtual characters [68], fostering more dynamic and diverse interactions. These advancements markedly enhance the authenticity and richness of virtual worlds, improving both visual fidelity and user engagement.

#### Transformers

**Fig. 4.**
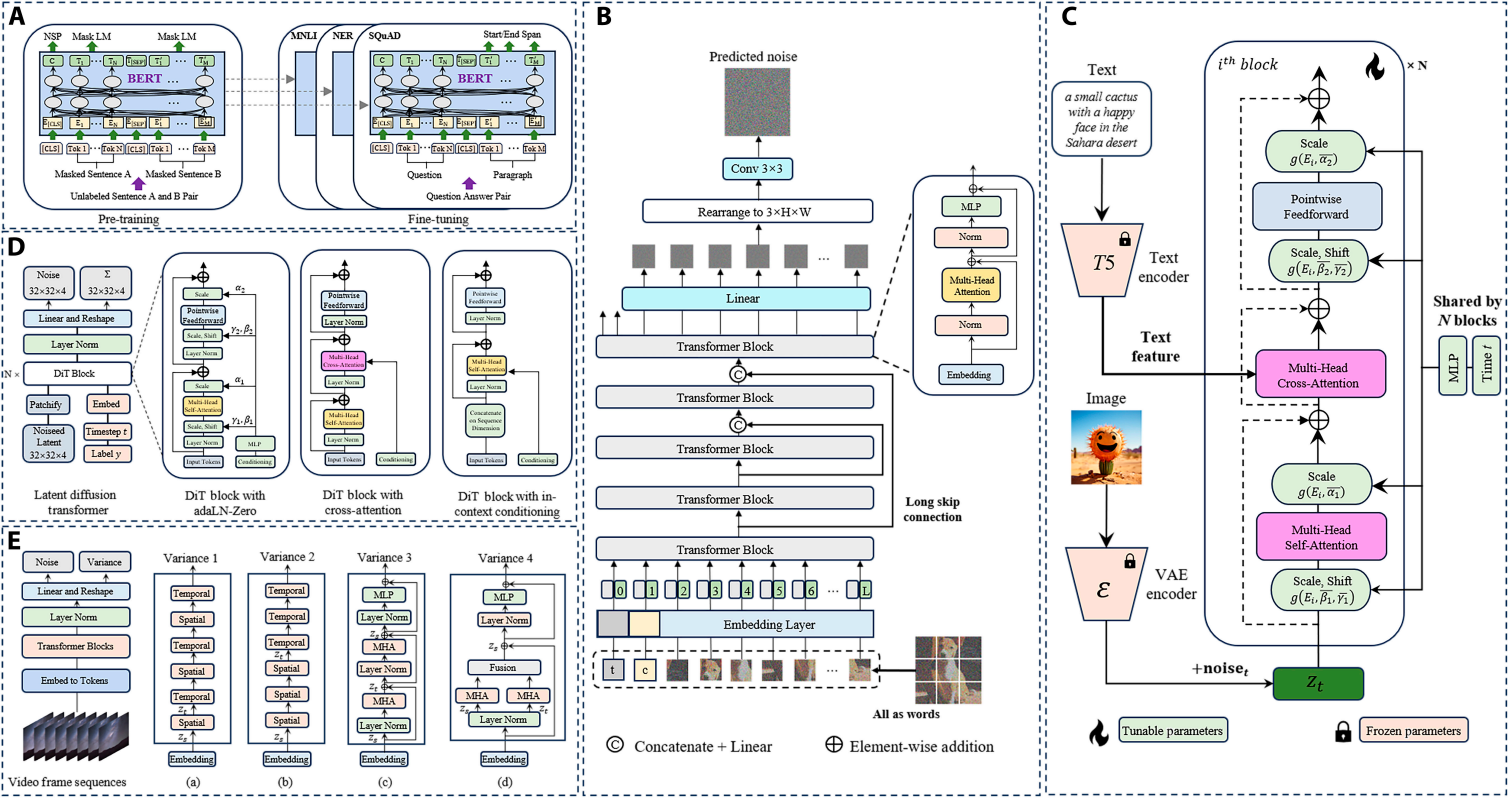
Overview of Transformer-based architectures for generative models. Zoom in for a better view. (A) Pre-training and fine-tuning procedures for Transformer-based BERT: architecture, parameter initialization, and special tokens [70]. (B) U-ViT architecture for diffusion models: tokenized inputs and long skip connections for efficient learning [71]. (C) PixArt-α architecture: cross-attention module for textual conditions and shared adaLN parameters for time efficiency [73]. (D) Diffusion techniques such as cross-attention mechanisms and spatiotemporal token extraction to enhance performance and output quality. The increasing reliance on Transformers for generating high-resolution, large-scale content underscores their transformative influence on generative AI, solidifying their position as a driving force in the field. Transformer (DiT) architecture trains conditional models by processing input latents with DiT blocks, incorporating adaptive layer normalization, cross-attention, and extra tokens, with adaptive layer normalization yielding the best performance [72]. (E) Latte pipeline for video generation: spatiotemporal information capture with Transformer variants and VAE simplification [74].

Transformers have become a transformative force in the field of GAI, greatly enhancing the capabilities of neural networks across a variety of domains. Initially introduced by Vaswani et al. [69], the Transformer architecture revolutionized NLP tasks such as machine translation and language generation, largely due to its innovative attention mechanism. We have compiled several transformer variants used for text/image/video generation and processing in Fig. 4 [70–74]. Unlike traditional models, Transformers are capable of modeling long-range dependencies by using self-attention and multi-head attention, enabling them to capture complex global relationships within sequences. The exceptional adaptability of transformers has made them the foundational technology behind the current state-of-the-art generative applications. Models like generative pre-trained transformers (GPT) [75] and bidirectional encoder representations from transformers (BERTs) [70] exemplify this capability, delivering highly coherent and contextually aware outputs across both textual and multimodal formats. This versatility highlights their pivotal role in advancing state-of-the-art generative systems.

In recent years, Transformers have expanded their influence beyond NLP, establishing dominance in computer vision and multimodal AI, where their scalability and ability to model complex patterns have led to breakthroughs in image and video generation. For instance, prior to the development of U-vision transformer (ViT) [71] and diffusion transformer (DiT) [72], state-of-the-art generative models for image synthesis relied on convolutional U-Net architectures. The integration of Transformer blocks into generative frameworks, as seen in U-ViT, enabled the treatment of all inputs as tokens while leveraging long-range connections, facilitating deeper and more efficient learning. DiT further advanced this by adopting ViTs as the core architecture for image generation, demonstrating their robustness and scalability in addressing intricate generative tasks. Innovations like PixArt-α [73] and Latte [74] continue to push the boundaries of Transformers in image and video synthesis, employing advanced cross-attention mechanisms, spatio-temporal token extraction, and hierarchical feature representations to significantly enhance generation fidelity, temporal coherence, and scalability for high-resolution content creation.

#### Mamba

**Fig. 5.**
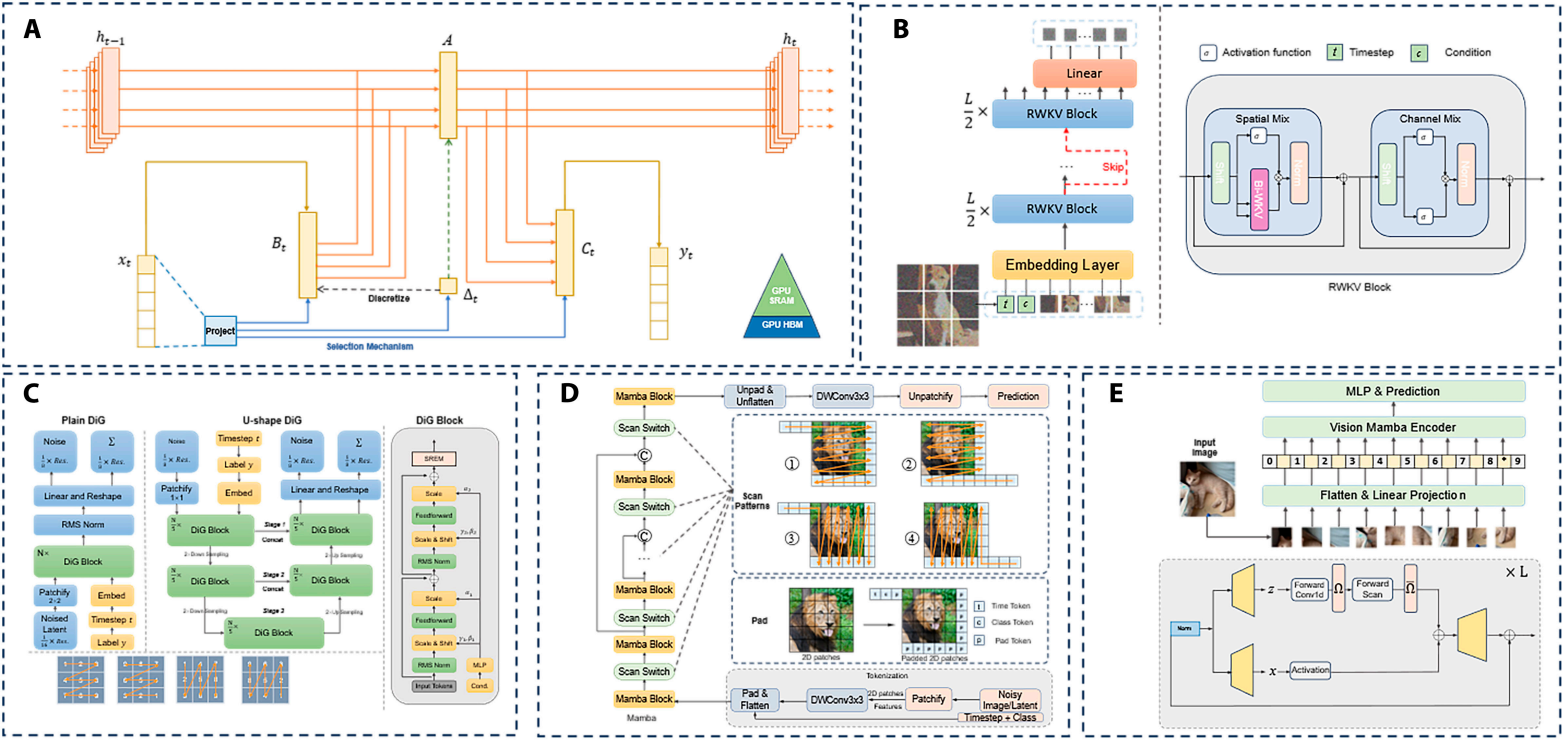
Advances of Mamba architectures for generative models. Zoom in for a better view. (A) Structured SSMs with input-dependent dynamics and hardware-efficient state expansion [80]. (B) Diffusion models with Bi-RWKV layers for spatial and channel mixing [81]. (C) Overview of DiG models: plain, U-shape, and block structures with SREM control [82]. (D) Noise prediction framework with patch-wise features and Mamba blocks in diffusion model [83]. (E) ZigMa: position-aware backbone with single-scan Mamba blocks in diffusion model [84].

Although Transformer-based GAI models have achieved exceptional performance, they face challenges when handling long-sequence generation tasks due to the quadratic computational complexity of the attention mechanism. This leads to substantial computational demands, especially when applied to high-resolution image synthesis or video generation tasks. However, recent developments in state-space modeling (SSM) [76,77] have introduced innovative strategies that effectively balance computational efficiency with model flexibility. Techniques such as those in Refs. [76,78,79] have proven highly effective across various tasks and modalities, particularly in managing long-term dependencies within sequential data. Building on these advancements, Mamba [80] was introduced, which integrates SSM principles with hardware-aware optimization techniques to simplify training and inference, thereby improving performance. We have appropriately summarized and presented the applicability of Mamba’s architecture and its various variants in text, image, and video generation and processing tasks in Fig. 5 [80–84]. Building on these advancements, the Diffusion Mamba (DiM) [83] model leverages Mamba as its backbone to enable high-resolution image generation. A key innovation in DiM lies in its enhancement of traditional unidirectional patch causality by incorporating an alternating scanning mechanism in the Mamba block, spanning 4 directional passes. To mitigate spatial continuity challenges associated with Mamba’s scanning technique, ZigMa [84] introduces an inductive bias specifically designed to ensure seamless continuity in 2D image applications. This methodology extends naturally to video generation, where ZigMa employs a spatiotemporal decomposition approach to handle 3D sequences, fostering more realistic motion and temporal consistency in video outputs.

To demonstrate the computational efficiency of Mamba, we compared the performance and parameter count of a Mamba-based architecture with Transformer-based and CNN-based architectures in an object tracking task based on OSTrack.

In addition to the aforementioned generative models, recent innovations in architectures for image and video generation have also emerged. For example, Diffusion-RWKV (receptance weighted key value) [81] integrates the RWKV [85] architecture as a backbone, utilizing temporal and channel mixing sub-blocks within its residual blocks. This design markedly improves upon traditional recurrent neural network (RNN) architectures by enabling parallelized computations during training and enhancing efficiency, particularly through the use of linear attention mechanisms. Additionally, DiG [82] introduces the Diffusion gated linear attention (GLA) [86] model, which incorporates a GLA Transformer to achieve exceptional training efficiency and optimized GPU memory utilization, making it highly effective for high-resolution image generation tasks.

To illustrate the computational efficiency of Mamba, we compared the performance and parameter counts of Mamba-based architectures with Transformer-based and CNN-based architectures on the OSTrack-based [87] tracking task. For a fair comparison, all methods were trained and evaluated on the large-scale event-based tracking dataset EventVOT [88], which contains 841, 18, and 282 videos in different subsets, respectively. The CNN-based methods include TrDiMP [89], ToMP 50 [90], DiMP 50 [91], PrDiMP [92], and ATOM [93]; the Transformer-based methods include HDETrack [88], AiATrack [94], STARK [87], TransT [95], MixFormer [96], and SimTrack [97]. We used 3 widely adopted evaluation metrics for comparison: success rate (SR), precision (PR), and normalized precision (NPR). Detailed experimental results are summarized in Table 1. It should be pointed out that all results are sourced from published papers. As shown in Table 1, CNN-based methods leveraging ResNet50 [98] generally have larger parameter counts but achieve slightly lower performance. Transformer-based methods demonstrate strong performance but require a substantial number of parameters. When replacing the ViT [99] backbone with Mamba, SR improves slightly, while PR and NPR show marginal decreases; however, the parameter count is significantly reduced to just 4.1M. These findings lead to the conclusion that Mamba offers high computational efficiency, achieving state-of-the-art performance with a minimal parameter count. This makes it a promising backbone, especially for handling large-scale 3D data, where its advantages become even more pronounced.

**Table 1.** Comparison between different methods on the EventVOT dataset. Bold formatting highlights the best computational efficiency of the Mamba architecture.

Tracker	Source	Backbone	SR	PR	NPR	Params (M)
TrDiMP	CVPR21	ResNet50	39.9	34.8	48.7	26.3
ToMP50	CVPR22		37.6	32.8	47.4	26.1
DiMP50	ICCV19		52.6	51.1	67.2	26.1
PrDiMP	CVPR20		55.5	57.2	70.4	26.1
ATOM	CVPR19		44.4	44.0	57.5	8.4
HDETrack	CVPR24	ViT	57.8	62.2	73.5	92.1
AiATrack	ECCV22		57.4	59.7	72.8	15.8
STARK	ICCV21		44.5	39.6	55.7	28.1
TransT	CVPR21		54.3	56.5	68.8	18.5
MixFormer	CVPR22		49.9	49.6	63.0	35.6
SimTrack	ECCV22		55.4	57.5	69.9	57.8
OSTrack	ECCV22	ViT-B	55.4	60.4	71.1	92.1
		ViT-S	52.0	53.2	66.8	54.3
		Vim-S	55.6	59.1	70.4	**4.1**

These advancements in AIGC are poised to significantly accelerate the development of the Metaverse. By enabling the creation of high-resolution, realistic, and contextually rich virtual environments, these technologies can enhance immersive experiences. Efficient video generation and real-time rendering capabilities will support dynamic, interactive worlds, while optimized memory utilization and computational efficiency will make large-scale Metaverse applications more accessible. Together, these innovations will drive the creation of more engaging, lifelike, and scalable virtual ecosystems.

### Comparative analysis of generative models for Metaverse applications

To systematically compare the performance of different generative models in Metaverse applications, this paper summarizes the strengths, weaknesses, and typical use cases of GANs, VAEs, diffusion models, and Transformers in this section. In addition, we further discuss the computational challenges associated with AIGC models and the limitations of current AR/VR hardware in supporting real-time, high-quality content generation. Finally, we analyze the performance of various generative models in terms of realism, coherence, and user engagement.

To provide a clearer understanding of the strengths, limitations, and practical considerations of different generative models in the context of Metaverse applications, we conducted a comprehensive comparative analysis. Specifically, to systematically compare generative models for Metaverse applications, we evaluate GANs, VAEs, diffusion models, and Transformers across quantifiable dimensions: latency, fidelity, controllability, multimodal capability, edge deployability, and Metaverse suitability. Table 2 synthesizes both quantitative benchmarks (FID [Frechet Inception Distance], latency, and Video Random Access Memory [VRAM]) and qualitative capabilities to highlight critical trade-offs. It highlights the trade-offs when deploying these models in resource-constrained yet interaction-intensive environments. For instance, GANs achieve domain-specific high fidelity (FID 2.5 to 4 on Flickr-Faces-HQ [FFHQ]) and sub-100-ms latency, enabling real-time applications like avatar generation, but lack multimodal support. Diffusion/Transformers offer superior controllability (e.g., ControlNet and prompt-based layout) and multimodal generation (text → image/video), but require >10 GB VRAM and suffer high latency (0.8 to 10 s). Edge deployment is viable only for GANs/VAEs (<8 GB VRAM), while Transformers demand cloud-scale resources (>24 GB VRAM). Overall, while diffusion/Transformer models enable complex multimodal generation, their high latency (>0.8 s) and hardware demands (>10 GB VRAM) limit real-time deployment. Conversely, GANs/VAEs support edge devices but lack open-domain flexibility. These constraints define their roles in the Metaverse stack: GANs/VAEs for interactive elements, and diffusion/Transformers for non-real-time content creation.

**Table 2.** Comparative evaluation of generative models for Metaverse content creation

Model type	Representative models	Latency (↓)	Fidelity (↑)	Controllability	Multimodal capability	Edge deployability	Suitability for metaverse scenarios
GANs	StyleGAN 3, BigGAN	30–70 ms (512^2^, A100)	FID: ~2.5–4 (FFHQ/CIFAR-10)	Latent interpolation	Image-only	<8 GB VRAM (INT8 quantized)	Virtual avatars, NPC faces, fashion try-on
VAEs	β-VAE, VQ-VAE-2	<100 ms (512^2^, A100)	FID: 28–32 (ImageNet-1k)	Basic attribute editing	Image-only	<4 GB VRAM (mobile GPU)	3D mesh encoding, texture compression
Diffusion models	Stable Diffusion 3, LDM	0.8–2.5 s (512^2^, A100)	FID: 5.5–6.5 (COCO val2017)	ControlNet + Inpainting	Text → image/video	10 GB VRAM (FP16 required)	Environment concept art, dynamic texture synthesis
Transformers	DALL·E 3, Imagen, Make-A-Video	2–10 s (512^2^, A100)	FID: 8–12 (COCO val2017)	Prompt + Layout control	Text → image/video/audio	>24 GB VRAM (multi-GPU)	Narrative scene generation, interactive storytelling

Table 3 presents a quantitative evaluation of representative models across distinct generative tasks: e.g., StyleGAN 3 [100] (unconditional/category-conditioned), VQ-VAE-2 [101] (text-to-image), Stable Diffusion 3 [102] (text-to-image), DALL·E 3 [100] (text-to-image), and Make-A-Video (text-to-video). Metrics include domain-specific FID/Fréchet Video Distance (FVD) scores [103], CLIP (Contrastive Language-Image Pre-Training) scores [104], latency, parameter size, and memory footprint. These measurements reveal critical deployment trade-offs: While DALL·E 3 and Stable Diffusion 3 achieve comparable high CLIP scores (~0.31) on open-domain COCO benchmarks, they require >10 GB VRAM and >1 s latency. In contrast, StyleGAN 3 and VQ-VAE-2 demonstrate domain-optimized efficiency with sub-100-ms inference and <8 GB VRAM requirements—making them suitable for edge deployment—though their low FID scores (StyleGAN 3: ~3.0 on FFHQ faces; VQ-VAE-2: 31.1 on ImageNet) reflect specialized rather than general-purpose capabilities.

**Table 3.** Performance and applicability of GANs, VAEs, diffusion models, and Transformers in Metaverse scenarios

Property	Evaluation benchmark (FID/FVD)	Representative	FID↓	CLIP score↑	Latency↓	Params (B)	Memory	Metaverse suitability
Unconditional/category-conditioned	FFHQ (human faces)	StyleGAN3	~3.0	N/A	<100 ms	~0.02	6–8 GB (single GPU)	Avatars, face edits
U0 text-to-image	ImageNet	VQ-VAE-2	31.1	N/A	<80 ms	0.1–0.3	<4 GB (single GPU)	Texture compression
	COCO (30K)	Stable Diffusion 3	~6.0	~0.31	~1,000–1,500 ms	~2.0–2.5	10–12 GB (A100 preferred)	High-fidelity images
	COCO (30K) or equivalent	DALL·E 3 (Transformers)	~10.39	~0.31	~2,000–3,000 ms	~12	≥20 GB (A100 preferred)	Creative design, storytelling
Text-to-video	UCF-101 (FVD)	Make-A-Video (diffusion)	~18.5 (video FVD)	N/A	~5–10 s (per clip)	~5.3	>24 GB (multi-GPU)	Not real time capable yet

We provide a quantitative summary of the resource requirements of state-of-the-art models for both image and video generation tasks. Table 4 details the total parameter size, average floating-point operations (FLOPs), memory requirements, and inference time on standard high-end hardware.

**Table 4.** Summary of model size, computation, and memory needs for generative tasks. FLOPs are estimated per single prompt and generation, assuming standard decoding steps. All metrics are based on FP16 mixed precision with optimized inference backends (e.g., Hugging Face Accelerate and TensorRT). Sora’s statistics are based on available public demos and research speculation, as the model architecture and training details remain unpublished.

	Model	Task	Parameters	FLOPs per generation	VRAM requirement	Inference hardware	Inference time
Image generation models (512 × 512 output)	Stable Diffusion 3	Image generation (512 × 512)	~2.0–2.5B	~400–500 GFLOPs	~10–12 GB (FP16)	NVIDIA RTX 3090	~2–2.5 s
	DALL E 3	Image generation (512 × 512)	~12B	~1–1.2 TFLOPs	~20 GB	Normalize to A100	Not comparable
	Imagen 4	Image generation (512 × 512)	>10B	~1.5–2.0 TFLOPs	>24 GB (TPU v4/A100 equiv)	Google TPU v4	Not comparable
Video generation models	Make-A-Video	16 frames @ 256 × 256	~5.3B	>2.5–3 TFLOPs	>24 GB	NVIDIA A100	~20 s
	Sora	48–120 frames @ 1,280 × 720*	>10B (est.)	>10 TFLOPs	>40 GB (cluster)	Multi-GPU A100 cluster	>30 s

The data above highlights that even high-end GPUs like the NVIDIA A100 or RTX 3090 struggle to generate high-resolution content from AIGC models in real time. Consider the following points: Real-time AR/VR requires latency below ~16 ms per frame (i.e., >60 FPS), but current AIGC image generation takes seconds per frame even with hardware acceleration. Standalone AR/VR headsets (e.g., Meta Quest 3 and Apple Vision Pro) typically have <16 GB of shared memory and integrated mobile GPUs (e.g., Snapdragon XR2 Gen 2), which cannot support the inference of billion-parameter models. Generation models such as Sora or Make-A-Video demand TFLOP-scale compute, multi-GPU setups, and substantial memory overhead, making on-device generation infeasible without cloud assistance.

Therefore, real-time AIGC deployment in AR/VR environments is currently constrained by hardware limitations. Future work should explore lightweight model design (e.g., diffusion distillation, quantization, and knowledge distillation), streaming-based rendering architectures, and edge–cloud hybrid deployment strategies to make high-fidelity generative experiences feasible in immersive systems. In addition to inference complexity, training large AIGC models incurs significant monetary cost, primarily due to the extensive GPU hours required.

Table 5 provides an estimate of training cost based on available literature, industry disclosures, and cloud GPU pricing benchmarks (assuming FP16 training on NVIDIA A100, $1.5 to $3.0/hour per GPU).

**Table 5.** Estimated training costs and resource requirements for large-scale AIGC models

Model	Training data	Training GPU-hours (A100-equiv hours)^a^	Estimated cost (US$)^b^	Source reliability^c^	Key assumptions
Stable Diffusion 3	LAION-2B + proprietary^d^	~200–300K	~600K–900K	High (official partners)	$3/A100-hour; public disclosures
DALL·E 3	Licensed images + synthetic text	~1.2–1.8M A100 h	~3.6M–5.4M	Medium (analyst estimates)	$3/A100-hour; scaled from architecture
Imagen 3 (Google)	Curated internal dataset	~350K (TPU → GPU conv)^e^	~1.0M–1.4M	Medium (research pubs)	TPU v4 → A100: 1.4× efficiency; $2.85/TPU-hour
Make-A-Video (Meta)	2.3B video–text pairs	2.0–2.5M	~6M–7.5M	High (Meta publication)	$3/A100-hour; confirmed scale
Sora (OpenAI, est.)	Undisclosed video (>100M clips†)	>8–12M	>24M^f^	Low (speculation)	Extrapolated from output quality; no official data

^a^ All costs normalized to $3.00/A100-hour (on-demand cloud pricing).

^b^ ±20% variance possible from optimal cluster utilization.

^c^ Reliability tiers: High (official disclosure), Medium (peer-extrapolated), AND Low (speculative).

^d^ Stable Diffusion 3 costs reflect open-source efficiency gains (architectural distillation, LAION-2B curation).

^e^ TPU→GPU conversion: 1 TPU v4 core ≈ 1.4 A100s (MLPerf v3.0 inference benchmarks).

^f^ Sora estimate derived from minimum viable training scale for 720p/60s physics-rendered video.

As summarized in Table 5, training AIGC models requires multi-million dollar investment, often only affordable to large corporations or institutions with access to proprietary data and massive compute clusters. Such training is typically only feasible for large corporations or institutions with access to proprietary datasets and massive compute clusters. Additionally, the environmental impact of training billion-scale models raises sustainability concerns, particularly with respect to carbon emissions.

These combined constraints explain why current AR/VR platforms cannot support on-device generation or customization of high-fidelity AIGC content. To overcome these challenges, future research should focus on lightweight model design (e.g., quantization, knowledge distillation, and diffusion acceleration), efficient adaptation techniques (e.g., low-rank adaptation [LoRA] and adapter layers), and edge–cloud hybrid architectures that offload heavy computation to remote servers while maintaining low-latency user experiences. Such strategies are essential to making AIGC viable for real-time, immersive applications.

Table 6 provides a comprehensive evaluation framework comparing GANs, diffusion models, and Transformers across 3 critical dimensions: realism, coherence, and user engagement—with domain-specific quantitative benchmarks. For realism, GANs achieve domain-specific excellence (FID 2 to 4 on FFHQ faces), diffusion models lead in open-domain fidelity (FID 5 to 6 on COCO), while Transformers offer superior multimodal realism (CLIP score 0.30 to 0.33). In coherence evaluation, diffusion models demonstrate controllable consistency (FVD 250 to 300 for video), whereas Transformers excel in cross-modal alignment (Winoground score 45% to 55%, temporal FVD 180 to 220)—both significantly outperforming GANs’ limited image-level consistency. For user engagement, GANs enable real-time interaction (<100 ms latency, 4 to 8 GB VRAM) ideal for avatar applications, while diffusion and Transformer models trade higher latency for semantically rich content that drives deeper engagement despite demanding >24 GB VRAM. This structured analysis confirms GANs’ dominance in latency-sensitive tasks like avatar generation, diffusion models’ advantage in high-fidelity scene rendering, and Transformers’ superiority in cross-modal storytelling—with each architecture’s hardware constraints directly informing their Metaverse applicability.

**Table 6.** Comparative evaluation of generative model families across realism, coherence, and user engagement dimensions

Aspect	GANs	Diffusion models	Transformers
Realism	Domain-specific excellence • FID: 2–4 (FFHQ) • Precise texture rendering	Open-domain fidelity • FID: 5–6 (COCO) • CLIP score: 0.30–0.32	Multimodal realism • FID: 8–12 (COCO) • CLIP score: 0.30–0.33
Coherence	Image-level only •No cross-frame consistency • Limited semantic control	Controllable consistency • FVD: 250–300 (UCF-101) • Layout accuracy: 75%–85%	Cross-modal alignment • Winoground score: 45%–55% • Temporal FVD: 180–220
User engagement	Low latency enables interactive applications (e.g., face editing, avatars) (real-time interaction)	High-quality outputs attract engagement but suffer from inference latency (VRAM: 4–8 GB [INT8] quality vs. speed trade-off)	High creative potential and multimodal generation encourage active user input (requires >24 GB VRAM)

Overall, we systematically compare the capabilities of GANs, VAEs, diffusion models, and Transformers for Metaverse applications across key dimensions such as latency, fidelity, controllability, and deployability. Through both qualitative and quantitative analyses, we highlight their respective strengths, limitations, and hardware constraints.

### Generative models in transition: Comparative insights into GANs, VAEs, Diffusion, Transformers, and Mamba

In the domain of generative models (AIGC), architectures such as GANs, VAEs, diffusion models, Transformers, and Mamba represent distinct theoretical foundations and developmental trajectories in generation mechanisms. Each of these models approaches generative tasks from different perspectives—probabilistic modeling, sequence modeling, or energy-based modeling—forming unique systems with their own strengths and challenges.

GANs and VAEs embody fundamentally different probabilistic frameworks. GANs employ an adversarial setup rooted in game theory, where a generator network learns to map noise vectors to the data distribution, while a discriminator learns to distinguish between real and synthetic samples. The generator improves by receiving dynamic loss signals from the discriminator, ideally reaching a Nash equilibrium where the generated samples become indistinguishable from real ones. A major strength of GANs lies in their implicit, likelihood-free optimization, which enables the generation of perceptually high-quality outputs—particularly effective in synthesizing high-dimensional data such as images and audio. However, GANs suffer from notorious training instability, including mode collapse and vanishing gradients. The theoretical basis leans more on minimax optimization and divergences like Jensen–Shannon, rather than explicit probabilistic modeling.

In contrast, VAEs adopt a variational inference-based framework with an explicit probabilistic encoder–decoder architecture. The encoder maps inputs to a structured latent space constrained by a Gaussian prior (typically enforced via KL divergence), and the decoder reconstructs data from this compressed representation. This results in a smooth, continuous latent manifold, well-suited for controlled sampling, albeit often at the cost of blurry outputs due to trade-offs in the approximated posterior. VAEs excel in uncertainty modeling but are typically less flexible than GANs in generating high-resolution outputs. Architecturally, GANs tend to rely on deep convolutional networks to capture sharp, local features, while lacking inherent encoding capability. VAEs, on the other hand, provide principled latent representations at the expense of generative sharpness.

Diffusion models represent a fundamentally different generative paradigm. These models conceptualize generation as the reverse of a forward diffusion process that gradually corrupts data by adding noise, transforming it into a Gaussian distribution. The model learns the reverse denoising process to reconstruct the original data distribution, typically modeled as a Markov chain or via stochastic differential equations, as seen in DDPMs and SGMs. Architecturally, diffusion models often use U-Net backbones enhanced with residual connections and attention mechanisms to capture multi-scale features during denoising. One of their standout advantages is high sample quality and training stability, as they allow fine-grained control over the generative process. However, due to their iterative nature, inference is significantly slower compared to other models, posing a bottleneck for practical deployment. Recent advancements such as DDIM and latent diffusion aim to address this issue by accelerating the sampling process.

Transformers, initially designed for sequence modeling, have redefined generative modeling through their attention-based architecture. Theoretically, Transformers rely on self-attention mechanisms to model global dependencies in data, making them highly effective for capturing complex, long-range relationships. This is formalized through multi-head attention, which computes weighted interactions among all input tokens. In generative contexts, Transformers process tokenized inputs—such as image patches or text subwords—through stacked layers of multi-head attention and feedforward networks. This enables powerful context modeling and parallel training over long sequences, though it comes with quadratic computational complexity with respect to sequence length. Extensions such as DiT and PixArt-α adapt the Transformer architecture for image and video synthesis by tokenizing inputs and applying cross-attention for conditional generation. Unlike GANs or VAEs, Transformers are highly scalable and effective for multimodal and multitask scenarios. However, they are computationally expensive and memory-intensive, especially when handling high-resolution visual data.

Mamba introduces a novel architecture based on state space models, aiming to overcome the quadratic complexity bottleneck that Transformers face when processing long sequences. Unlike attention-based models, Mamba’s core innovation lies in its structured SSM layers, which efficiently model long-range dependencies with linear time complexity and sublinear memory growth. This makes it especially well-suited for long-context generation tasks. Mamba integrates input-dependent dynamics, allowing the model to selectively retain or forget information based on the current input, along with hardware-friendly state expansion mechanisms. To make Mamba viable for generative tasks like image synthesis, models such as DiM and ZigMa enhance the architecture with strategies like alternating scan patterns to maintain continuity in 2D spatial data and mitigate artifacts introduced by sequential scanning.

From a theoretical standpoint, Mamba strikes a balance between the stateful representation power of RNNs and the training efficiency of CNNs. While Transformers handle token interactions via explicit attention weights, Mamba captures dependencies implicitly through input-driven state evolution. This design trades some of the interpretability of global interactions for superior scalability, particularly valuable in dense generation tasks such as image and video synthesis. Key architectural innovations include selective state expansion and directional scanning strategies (e.g., zigzag patterns). Consequently, Mamba excels in balancing model expressiveness with computational efficiency, effectively bypassing the Transformer’s scaling issues. Its lightweight design contrasts sharply with the heavy computational demands of Transformers and diffusion models, positioning it as a promising and efficient alternative for scalable generative applications—especially those involving long sequences or high-resolution content—though its application in high-fidelity synthesis is still rapidly evolving.

In summary, these models each offer unique strengths in generative mechanisms and capabilities. VAEs emphasize structured latent spaces; GANs focus on perceptual realism; diffusion models prioritize stability and controllability; Transformers bring unmatched sequence modeling power; and Mamba charts a new path toward efficient, scalable generation. Their evolution reflects broader trends in generative modeling—from probabilistic to structural modeling, and from single-modal to multimodal generation. A key challenge for future models will be integrating the advantages of these diverse architectures to achieve a holistic balance of quality, efficiency, and controllability in generation.

### AIGC driving the future of content creation

Building upon foundational deep learning models, AIGC has made significant strides in text-to-image, text-to-video, and text-to-3D scene generation. In text-to-image synthesis, the integration of Transformers and diffusion models has enhanced both fidelity and efficiency, enabling high-resolution, semantically accurate image generation. In text-to-video generation, advancements in spatiotemporal modeling, diffusion techniques, and multimodal fusion have facilitated the creation of longer, more dynamic sequences with improved semantic consistency and physical plausibility. For 3D scene generation, the combination of NeRF, geometry-guided diffusion, and NeRF optimization has overcome limitations in sparse-view reconstruction, enabling high-fidelity, interactive 3D environments. With the continuous advancement in integrating AIGC with robotic technologies, robotics is transforming AIGC’s “digital creativity” into tangible “physical productivity”, while simultaneously driving the evolution of AI models through real-time data feedback loops. These breakthroughs not only streamline content creation but also lay the technical foundation for the development of the Metaverse.

#### Image generation

In the field of text-to-image generation, numerous novel generative paradigms and innovative technologies have emerged in recent years. OpenAI’s DALL-E [105] pioneered Transformer-based synthesis via a 2-stage framework: compressing images into 32 × 32 discrete tokens with VQVAE, then modeling text–image joint distributions via autoregressive Transformers, achieving zero-shot capabilities and highlighting computational scaling’s role. Guided Language to Image Diffusion for Generation and Editing (GLIDE) [106] validated classifier-free guidance for diffusion models, directly inspiring DALL-E2 [107], which combined CLIP embeddings with hierarchical diffusion to achieve 4× resolution gains (1,024 × 1,024). DALL-E3 [108] enhanced prompt adherence 2.3× via synthetic caption refinement, improving rare-object and spatial modeling. Google’s Imagen [109] leveraged frozen T5 [110] large language models (LLMs) with diffusion cascades, showing that language model scaling improved FID scores 37% vs. 12% for image models, while its 2-stage super-resolution reduced memory by 83%. Parti [111] used ViT-VQGAN tokenization and a 20B-parameter Transformer for autoregressive 1,024 px generation, outperforming diffusion models in compositional coherence by 19%. Muse [111] accelerated synthesis 10× via masked token modeling (12 steps vs. 128), with StyleDrop [112] capturing artistic styles via 60K-parameter tuning (94% user preference). Stable diffusion’s latent-space optimization revolutionized efficiency: compressing 512 px images into 64 × 64 tensors enabled 4.8× faster diffusion in 48D space, paired with dynamic CLIP–text fusion for precise attribute binding. Recent studies have redefined model interpretability and efficiency. Tang et al. [113] explored syntactic influences in diffusion models through DAAM (diffusion attentive attribution maps), mapping linguistic structures to pixel-level heatmaps to decode how grammar shapes image generation. Concurrently, Feng et al. [114] devised training-free structured guidance by modifying cross-attention layers, enabling precise attribute-object binding while preserving multi-attribute semantics. On the optimization front, Li et al. introduced Colossal-AI, a framework enhancing resource efficiency for large generative models. Building on this, Stability AI’s SD-XL [113] integrates an enhanced U-Net backbone with advanced conditioning techniques—such as resolution-aware embeddings—and a robust text encoder, achieving higher fidelity and scalability. These innovations highlight a trajectory toward hybrid architectures (diffusion + Transformers + LLMs), data-engineered precision, and efficient latent processing, paving the way for real-time, high-fidelity generation with granular user control.

The field of text-to-image generation is advancing toward a synthesis of multimodal intelligence, computational efficiency, and human-centric creativity. Future progress will likely hinge on unifying diffusion models, autoregressive Transformers, and LLMs into hybrid architectures that embed deeper semantic understanding while resolving persistent challenges in spatial reasoning and rare-object composition. Innovations in dataset engineering—such as synthetic caption refinement and grammar-aware annotation—could bridge the gap between textual precision and visual fidelity, particularly for complex scenes. Simultaneously, lightweight adaptation techniques like parameter-efficient fine-tuning and resolution-aware cascades promise to democratize high-quality generation, enabling real-time, interactive applications on consumer devices. Emerging interpretability frameworks, such as attention manipulation guided by linguistic structures, may unlock finer control over attribute binding and compositional logic. As computational optimizations in latent-space processing and adaptive compression mature, the boundary between photorealistic accuracy and artistic stylization will blur, fostering tools that dynamically adapt to user intent. These advancements, coupled with open-source ecosystems and ethical dataset practices, could redefine content creation in the Metaverse—ultimately enabling systems that transcend static image synthesis to become collaborative, context-aware partners in visual storytelling.

#### Video generation

The evolution of video synthesis technology has been driven by a series of iterative architectural innovations, accompanied by pivotal shifts in methodology. Early research by Vondrick et al. [115] pioneered a GAN-based spatiotemporal modeling architecture, introducing the separation of dynamic foreground elements from static backgrounds in both spatial and temporal dimensions. This groundbreaking work laid the foundation for subsequent studies, with Tulyakov et al. [116] proposing a recurrent framework to decompose motion embeddings from content representations, further enhanced by the introduction of 3D convolutional networks [117,118], which improved temporal consistency by 37% compared to 2D methods. Concurrently, Karras et al. [119] introduced a progressive refinement paradigm, generating high-resolution videos through iterative spatiotemporal upsampling.

The rise of Transformer technology revolutionized video generation strategies. Wu et al. [120] first integrated VQ-VAE [121] with 3D sparse attention mechanisms, enabling open-domain text-to-video synthesis. Subsequent research further advanced the field: Hong et al. [122] proposed a multi-frame-rate hierarchical training method to effectively align textual semantics with visual narratives, while Kondratyuk et al. [123] unified multimodal inputs (e.g., text, depth maps, and optical flow) into token sequences processable by LLMs, achieving 91% accuracy in complex scene transition tasks. The introduction of diffusion models marked another significant breakthrough in video synthesis. Ho et al. [124] extended 2D U-Net to 3D video denoisers and optimized them with pseudo-3D convolutional layers [125], reducing computational costs by 58%. Zhang et al. [126] proposed a hybrid architecture combining pixel-space initialization with latent upsampling, enabling the generation of 4K-resolution videos at 30 fps. Additionally, Blattmann et al. [127] emphasized the importance of high-quality dataset curation, with their filtered training corpus improving the realism metrics of generated videos by 42%.

Despite significant progress, the quality of generated videos still faces numerous challenges. Studies show that current systems exhibit perceptual discrepancies in 38% of generated sequences [128], particularly struggling to maintain physical plausibility in clips longer than 5 s. Furthermore, instruction misalignment manifests as cumulative semantic drift (0.9% per unit of prompt complexity), which traditional evaluation metrics like FVD [129] fail to detect effectively. The inadequacy of evaluation frameworks is also pronounced, as no automated framework has achieved a correlation greater than 0.72 with human assessments across 14 quality dimensions [130], highlighting the need for developing multimodal benchmarking systems, such as those integrating physics-aware validators [131] and causal relationship trackers. Recent research has proposed neural ODE (ordinary differential equations)-based temporal modeling methods [132] and the Phenaki model [133]—the latter capable of generating variable-length, high-quality videos from a series of open-domain text prompts—offering new possibilities for open-domain video generation and complex narrative tasks. However, practical applications still require further breakthroughs in long-range dependency modeling and efficient sampling techniques.

#### 3D scene generation

3D environments are foundational to digital media, gaming, and immersive platforms, but their creation typically requires multi-view data, extensive computational resources, and expert intervention, making the process costly. To address these challenges, researchers have proposed various innovative approaches. The dominant paradigm, “external-focused synthesis,” simulates real-world observation by capturing scenes from internal viewpoints. For example, Text2Room [134] generates room-scale 3D meshes from text, Text2NeRF [135] combines NeRF with diffusion models for zero-shot scene synthesis, RoomDreamer [136] enhances structural coherence through geometry-guided diffusion, and FastScene [137] achieves rapid reconstruction using 3D Gaussian splatting. Additionally, alternative methods like MAV3D [138] and 4D-fy [139] focus on dynamic scene generation, while perpetual view exploration frameworks such as SceneScape [140] and Vivid-Dream [141] enable limitless scene navigation. Object-compositional approaches like CompoNeRF [142] and Set-the-Scene [143] treat scenes as modular components, supporting flexible editing and synthesis.

Emerging technological advancements, exemplified by innovations like Apple Vision Pro, are propelling the development of the Metaverse from the proof-of-concept phase into a new era characterized by practical applications, heightened immersion, and multimodal integration. The synergistic combination of spatial computing and AIGC facilitates the evolutionary transition from a “virtual world” to a symbiotic coexistence of virtual and physical realities. Notably, Vision Pro’s Room Mesh capability leverages AIGC techniques to generate interactive 3D spatial models in real time, enabling users to directly “grasp” and position virtual objects on physical surfaces. This system achieves near-natural interaction by replacing traditional controllers with advanced biometric recognition (eye-tracking and gesture control), thereby approaching the critical threshold of seamless human–computer interface. This technological progression demonstrates 3 significant paradigm shifts: the realization of dynamic spatial computing through AI-generated 3D reconstruction, the emergence of intuitive interaction modalities that minimize cognitive load, and the creation of persistent hybrid environments where digital and physical elements maintain coherent spatial relationships.

These developments not only enhance user presence and operational fidelity but also establish foundational infrastructure for next-generation mixed reality applications across professional domains including industrial design, medical visualization, and collaborative engineering. The technical architecture exemplifies how contemporary AIGC implementations are overcoming historical limitations in real-time rendering latency and interaction naturalness that previously constrained Metaverse adoption.

Looking ahead, 3D environment generation technologies will evolve toward hybrid neural-explicit representations (e.g., NeRF–mesh fusion), physics-aware constraints, and lightweight on-device optimization. By integrating multimodal inputs (e.g., sketches, voice, or LLMs), the barriers to 3D creation are expected to be further lowered, bridging the gap between creative ideation and technical execution. These advancements will not only reduce reliance on expert knowledge but also markedly enhance the realism and interactivity of virtual environments, driving further progress in digital media and immersive experiences.

#### Robotics–AIGC

Robotics is emerging as a critical interface between AIGC and the physical world, serving as an essential conduit for bidirectional interaction through embodied AI systems. This paradigm enables the translation of digital content into physical actions while simultaneously incorporating real-world feedback into AI training loops, thereby establishing a closed-loop learning framework.

**Fig. 6.**
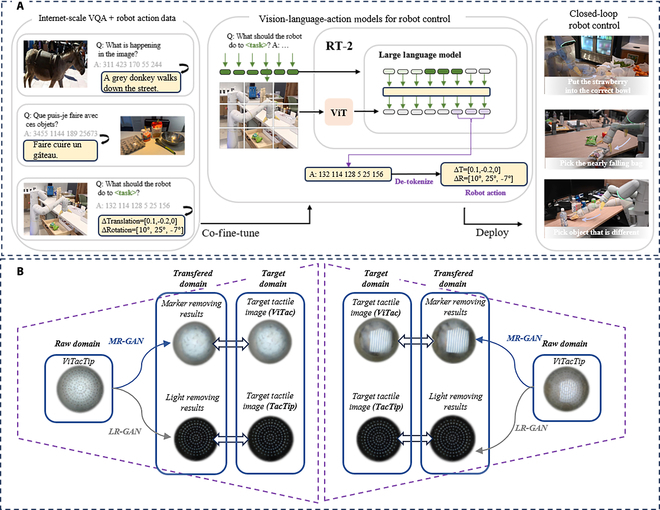
The collaborative development of robotics and AIGC. Zoom in for a better view. (A) RT-2 [144] overview. (B) Examples of modality conversion when applying MR-GAN and LR-GAN to cube-like objects with complex textures [145].

State-of-the-art multimodal LLMs (e.g., GPT-4V and PaLM-E) demonstrate remarkable capabilities in parsing natural language instructions (such as “organize the cluttered table”) and generating executable robotic action sequences (grasping, sorting, and placing). As shown in Fig. 6A, the Google RT-2-X [144] model exemplifies this advancement by integrating vision-language models with robotic control systems, achieving zero-shot execution of novel tasks (e.g., “hand the cola to the waving person”) without prior specific training.

Conversely, robotic systems function as physical “sensors” for AIGC frameworks, with their integrated force, tactile, and visual sensors continuously capturing real-time physical interaction data to enhance AIGC model training. The ViTacTip [145] tactile sensor shown in Fig. 6B, for instance, employs visual signals to indirectly measure tactile information, thereby improving AI models’ understanding of physical interactions. Motion data acquired by Tesla’s Optimus humanoid robot in real-world environments contributes to training more versatile embodied AI models capable of dynamic scene understanding.

This symbiotic relationship demonstrates how robotics transforms AIGC’s “digital creativity” into tangible “physical productivity” while simultaneously driving continuous AI model evolution through real-time data feedback loops.

### Connection of AIGC to the Metaverse

The development of the Metaverse confronts several fundamental challenges, including inefficient content production, suboptimal interactive experiences, constrained physical simulations, and limited personalization capabilities. AIGC fundamentally addresses the content scalability bottleneck in Metaverse construction by revolutionizing production pipelines through GAI models.

AIGC effectively resolves computational and cost barriers in 3D asset generation. Traditional 3D modeling requires manual creation of high-polygon models, whereas NeRF technology generates implicit neural representations of 3D scenes directly from 2D image inputs, reducing modeling time from hours to minutes. Multilayer perceptrons learn mapping functions from 3D spatial coordinates to color and density values $F\left(x,y,z,\theta ,\phi \right)\to \left(\mathrm{RGB},\sigma \right)$, eliminating the need for explicit mesh structures. Stable Diffusion 3D [146] bypasses conventional Blender/Maya pipelines by directly generating textured 3D models from text prompts using diffusion models. NVIDIA Omniverse benchmarks demonstrate that AIGC tools can improve 3D scene production efficiency by orders of magnitude—scenes requiring 1 week through traditional methods can now be generated within minutes using AI.

AIGC ensures multimodal content consistency through unified vector space mapping. Technologies like OpenAI’s CLIP [147] embed text, images, and 3D models into a shared latent space, maintaining semantic coherence across modalities. Advanced systems like Google’s Parti [148] employ jointly trained text–image–3D diffusion models with cross-attention mechanisms to synchronize multimodal content generation. These technological advancements essentially transform Metaverse content production from manual workflows to AI-driven pipelines, where scalability is no longer constrained by human resources but rather determined by computational power and data availability-constituting the foundational infrastructure for the “infinite Metaverse” paradigm in the Web3.0 era.

The Metaverse scene generation encompasses the large-scale creation of virtual environments, including buildings, landscapes, and interactive objects. AIGC plays a crucial role in the construction and evolution of the Metaverse by addressing content scarcity and enabling the rapid creation of virtual scenes, objects, characters, and events. This capability accelerates the replication of the physical world within digital environments and promotes the unlimited expansion of virtual content. AIGC technology expedites the creation of diverse virtual scenes, characters, buildings, and products, facilitating user interaction and engagement within the virtual realm. AIGC enhances architectural design efficiency and provides dynamic, personalized, and interactive experiences in the Metaverse. Additionally, AIGC enables the real-time and personalized generation of game elements, unique gameplay mechanics, and adaptive dialogues and interactions in virtual social contexts. These capabilities enhance user immersion by creating vibrant, context-specific experiences tailored to individual preferences. For instance, text generation technology, powered by NLP, can be applied in both non-interactive and interactive scenarios. Non-interactive text generation includes tasks such as summarization, title generation, text style transfer, and article generation, while interactive text generation is applied in chatbots and text-based interactive games. The development of the Metaverse confronts several fundamental challenges like AI Dungeon [149]. The integration of interactive text generation technology has become increasingly vital for enhancing the Metaverse experience, as it markedly improves interaction realism and content diversity through dynamic response to user inputs, environmental narrative generation, and virtual character behavior simulation. Recent advancements in AIGC for interactive text applications have demonstrated substantial progress, particularly exemplified by 2 groundbreaking developments. The May 2024 release of OpenAI’s GPT-4o represents a major leap forward with its unified multimodal architecture that enables seamless end-to-end processing across text, speech, images, and video modalities, while simultaneously achieving remarkable latency reduction to 232 ms in voice interactions through innovative neural compression techniques. Complementing this advancement, NVIDIA’s Genesis technology within the Omniverse ecosystem has revolutionized physical simulations by replacing conventional rigid-body dynamics computations with neural network-based approaches, resulting in a threefold improvement in the authenticity of virtual object interactions. These technological breakthroughs collectively address 2 fundamental challenges in Metaverse development: ensuring continuous cross-modal interaction fidelity and maintaining physics-based behavioral authenticity, thereby pushing the boundaries of what is achievable in virtual environment realism and user engagement. The convergence of these innovations marks a significant milestone in creating more immersive and responsive virtual worlds that better bridge the gap between digital and physical experiences.

In the realm of image generation, AIGC supports both image editing/modification and autonomous image generation. Image editing includes tasks like super-resolution, repair, face replacement, and background removal, while autonomous image generation uses deep learning and GANs to generate images based on descriptions, such as DALL-E [105], which can create high-quality images from user-provided descriptions and demonstrate exceptional detail handling capabilities.

Video generation technology further automates content creation, allowing for the efficient production of dynamic and high-quality video content from simple text prompts. Products like VEED [150] can automatically generate short videos based on textual input. Additionally, research projects like Make-A-Video [58] and Glia-Cloud [151] have achieved text-to-video generation. For instance, OpenAI’s “Sora” model can generate up to 60-s high-definition videos through text commands, with detailed backgrounds, multi-angle shots, and emotional characters [152]. This provides content creators with tools to bring ideas to life with lower costs and higher efficiency.

We can glimpse that emerging collaborative protocols bridge human creativity with generative precision—architects sketch concepts refined into physics-compliant 3D models via bounding-box-guided diffusion, while screenwriters trigger multi-angle cinematic previz through text–video synthesis. Crucially, neural ODE-based physics validation ensures that synthetic objects obey virtual world laws, forming a tripartite framework where human intent, AI generation, and digital physics co-evolve. These advancements dissolve traditional production silos, reimagining Metaverse content pipelines as open networks of human-AI co-creation. The endgame mirrors Neal Stephenson’s “Metaverse protocol”—a living digital civilization where every user, armed with natural language interfaces, becomes both consumer and creator, collectively weaving an ever-evolving tapestry of immersive experiences through seamless access to generative comp.

With the assistance of AIGC, the Metaverse is poised to shine in fields such as healthcare, education, psychology, and sociology. For example, in the healthcare sector, the Metaverse plays a transformative role, particularly in medical training and patient support.

#### Healthcare

With a rapidly aging population and a shortage of healthcare workers, the Metaverse as well as AIGC can also be used in healthcare, including telemedicine, clinical care, physical health, mental health, and more [153,154]. The biggest advantages of Metaverse are that it eliminates geographic limitations, enhances the traditional Internet in 3D, and makes the experience immersive through an immersive experience platform.

In particular, the Metaverse offers a broad spectrum of transformative use cases in healthcare, reshaping how care can be delivered, accessed, and experienced. AIGC enables the creation of realistic simulations, allowing practitioners to conduct virtual consultations, perform diagnostics, and even simulate surgical procedures with unparalleled precision. This capability eliminates geographical barriers, ensuring that individuals in remote or underserved regions have access to quality healthcare. These virtual patient consultations in the Metaverse could elevate otherwise staid telemedicine, allowing immersive 3D interactions between patients and physicians. Patients could also gain rehabilitation and physical therapy benefits from gamified exercises and remote monitoring within the Metaverse in AIGC-generated environments. In addition to physical health, mental healthcare has the potential to be revolutionized with Metaverse-based therapies that provide safe spaces for treating posttraumatic stress syndrome, anxiety, and depression.

Medical education becomes more immersive with anatomy explorations, virtual dissections, and simulated hospital environments within the Metaverse via AIGC. The Metaverse could also ultimately support remote surgery, chronic disease management, and real-time health monitoring through wearable devices that integrate access to these technologies.

Beyond standard healthcare, the Metaverse could also foster community care via virtual support groups for patients with rare or chronic illnesses and introduces therapeutic gaming for neurorehabilitation in comfortable and familiar AIGC generated environments. Additionally, drug development and clinical trials could be expedited through collaborative virtual simulations [155].

The Metaverse can also enhance palliative care by offering AIGC optimized immersive experiences for hospice patients, promotes fitness and preventative care with interactive virtual wellness programs, and prepares healthcare professionals for disaster response with virtual training. All in all, these applications collectively highlight the Metaverse’s capacity to create a more innovative, patient-centric, and accessible healthcare ecosystem [156].

The Metaverse, as an immersive virtual environment, enables new forms of interaction and training, such as VR-based social skills training for individuals with autism spectrum disorder [157] and posttraumatic stress disorder [158]. Although current Metaverse implementations have limitations in adapting to the needs of these patients, advancements in adaptive and personalized technologies, including deep learning and generative agents, are overcoming these barriers.

#### Psychology

In the field of education, the integration of AIGC with VR technologies has created unprecedented learning experiences. By constructing highly immersive virtual learning environments, this technology not only overcomes the physical limitations of traditional classrooms [159] but also drives 3 major innovations in educational models: First, the multisensory embodied learning experiences facilitated by VR/AR technologies markedly enhance knowledge acquisition efficiency in complex disciplines such as STEM. Second, AIGC-generated virtual classrooms effectively eliminate geographical barriers, providing equitable learning opportunities for underserved regions. Third, students and educators worldwide can engage in real-time collaboration within shared virtual spaces, realizing a truly borderless educational community. This innovative approach further extends to professional training, enabling high-risk industries like healthcare and aviation to conduct high-fidelity simulations in safe virtual environments.

From a mental health perspective, AIGC’s performance in the Metaverse is equally noteworthy. Research indicates that emotionally intelligent virtual spaces created by this technology can precisely address users’ psychological needs, demonstrating significant clinical value in alleviating conditions such as anxiety and depression. Through carefully designed therapeutic environments, guided meditation experiences, and supportive social interactions, AIGC provides an effective psychological regulatory space for modern individuals. However, this technological empowerment also brings notable social risks. Recent studies suggest that excessive reliance on virtual experiences may lead to the deterioration of real-world social skills [160], while blurred boundaries between virtual and physical realities could trigger identity confusion. Moreover, the anonymity of the Metaverse may exacerbate behavioral misconduct [161].

Addressing these challenges requires a comprehensive strategy. At the technical level, intelligent usage monitoring systems—including adaptive time reminders and content filtering mechanisms—should be developed. Ethically, design principles centered on promoting holistic human development must be established. Socially, digital literacy education should be strengthened to cultivate healthy balance between virtual and physical engagement. Only through such multifaceted approaches can the healthy development of AIGC technology in the Metaverse be ensured, ultimately realizing its social value in advancing educational equity and psychological well-being.

#### Sociology

From a sociological point of view, AIGC has a profound impact on the social structure in the Metaverse. The wide application of AIGC enables users to ship and own unique virtual assets, such as digital artifacts, virtual real estate, and so on. The difference in the value of these assets may lead to a new social stratification in the meta-universe, forming a class structure similar to that of the real society. Meanwhile, with the development of AIGC technology, many new professions have emerged in the Metaverse, such as virtual architects and digital fashion designers. The rise of these occupations has changed the traditional classification of occupations and enriched the diversity of social roles [162]. Secondly, AIGC has also had a certain impact on social interaction modes: the deepening of human–computer interaction, the virtualization of social networks, and the diversity of social interactions [163,164]. AIGC technology has enabled virtual characters to have higher intelligence and emotional expression capabilities, and users’ interactions with these virtual characters have become more natural and in-depth, and may even develop an emotional connection similar to interpersonal relationships. Similarly, users can interact and participate in a variety of virtual activities through the created virtual identities, generating personalized social content according to user preferences and behaviors, changing the traditional mode of social interaction, and enriching the social experience.

In addition to its impact on social structure and interaction, AIGC in the Metaverse has the potential to promote environmental sustainability, particularly through the development and use of digital assets in lieu of physical ones. One notable example is the field of fashion, where “Metaverse fashion” has already emerged as a potential alternative to traditional clothing, at least to some degree—particularly for the generations that see clothing as a signaling opportunity via online social media.

To wit, digital garments, designed and traded entirely within virtual environments and worn by avatars in the Metaverse, can satisfy consumers’ desire for self-expression and novelty without the environmental costs associated with physical fashion production. Unlike traditional fashion, which often relies on resource-intensive manufacturing processes, generates significant textile waste, and contributes to water pollution and carbon emissions, digital fashion offers a waste-free, sustainable option. Users can experiment with endless styles and designs in the Metaverse, showcasing their creativity and identity through virtual avatars without generating tangible waste or requiring mass production. This shift not only reduces the ecological footprint of the fashion industry but also addresses the issue of fast fashion, which has long been criticized for its unsustainable practices. By providing an environmentally friendly alternative that still fulfills the cultural and social functions of fashion, the Metaverse has the potential to reshape consumer habits and promote a more sustainable approach to self-expression and creativity [165].

Ultimately, the application of AIGC algorithms in the Metaverse introduces several challenges and risks that must be addressed to ensure ethical and safe usage. One major concern is the potential for data bias and discrimination during the training phase. While AIGC enhances user immersion, it also increases vulnerability to adversarial attacks, where malicious training samples or gradient poisoning could perpetuate biases in models. This can worsen social inequalities and marginalize certain groups. Additionally, the extensive data requirements for training AIGC models—often including sensitive personal information—raise significant data security and privacy concerns. Without robust privacy protections, the risks of unauthorized access, data breaches, and misuse of user data are heightened.

To mitigate these risks, several strategies should be implemented. Responsible AI practices must be adopted, focusing on ethical AI development frameworks that prioritize bias mitigation and fairness in both training data and model outcomes [166]. Supervised learning techniques can be employed to identify and eliminate biases within training datasets, helping reduce the potential for social inequality. Privacy-enhancing technologies, such as federated learning, should be utilized to train AIGC models without centralizing sensitive user data, minimizing privacy risks. Additionally, strong data security measures, including encryption, secure data transmission, and access controls, are essential to safeguard user data. Comprehensive security protocols, such as data encryption, authentication, and network security, should be introduced to protect user privacy in the Metaverse. Regular updates to security protocols are also necessary to adapt to evolving threats and incorporate the latest technological advancements.

#### From technology to practice: AIGC’S industrial footprint

Building upon these technical advancements, a growing number of enterprises and research institutions have begun to integrate AIGC into real-world applications. These implementations span multiple sectors—including healthcare, education, industry, and entertainment—demonstrating not only the practical viability of AIGC but also its transformative potential across diverse domains. The following representative case studies illustrate how the synergy between foundational models and domain-specific adaptations is reshaping content creation, simulation, and interaction in the Metaverse.

**Fig. 7.**
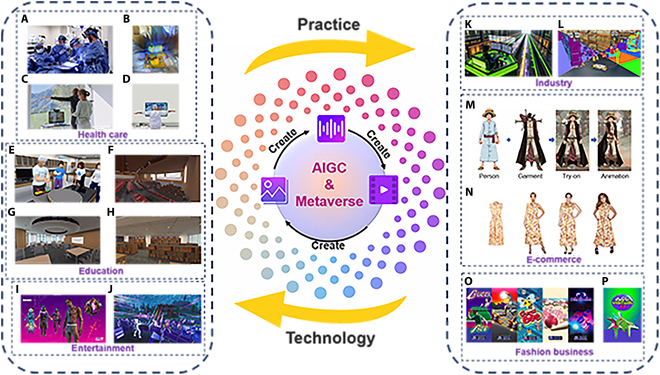
The practical applications of the Metaverse. (A and B) SyncAR system by surgical theater: AR-enhanced multimodal navigation for tumor boundaries in intraoperative microscopic view. (C and D) VR-augmented multimodal neurorehabilitation: MindMaze’s digital therapy platform. (E to H) Virtual reality metaversity developed by Victory XR: a new paradigm for VR education. (I and J) Epic games virtual concert: showcasing cross-dimensional performance innovation. (K and L) NVIDIA omniverse × OpenUSD alliance: standardizing physics-accurate 3D ecosystems for the Metaverse era. (M and N) AI–fashion convergence: Alibaba’s GAN-powered “outfit anyone” virtual fitting revolution. (O and P) GUCCI’s GOOD GAME Metaverse: retro-gaming, NFT exclusivity, and the rebirth of luxury storytelling.

The integration of AIGC into the Metaverse shown in Fig. 7 has demonstrated transformative potential across diverse sectors, supported by empirical evidence of its efficacy. In healthcare, platforms like Surgical Theater [167] leverage diffusion models and NeRF to generate high-fidelity 3D patient organ models, markedly reducing preoperative planning time and lowering intraoperative complication rates in complex neurosurgical procedures, as evidenced by clinical trials at the Cleveland Clinic. Similarly, Mind Maze [168] employs GANs to create personalized neurorehabilitation environments, accelerating motor function recovery in stroke patients, validated through Fugl-Meyer assessments in Swiss clinical studies. NVIDIA CLARA’s GAN-based synthetic medical imaging [169] addresses rare disease data scarcity, markedly improving brain tumor segmentation accuracy, while Johns Hopkins University’s diffusion model-driven 3D anatomical simulations for surgical training have streamlined procedural efficiency and reduced error rates in resident assessments.

In education, platforms such as Victory XR [170] utilize stable diffusion to automate the generation of immersive historical simulations, enhancing student performance and course completion rates in U.S. university pilots. Meta’s collaboration with Stanford University demonstrated that diffusion-generated 3D historical environments (e.g., ancient Rome) markedly elevated student knowledge retention and engagement. Similarly, Peking University’s Transformer-based virtual language coaching system improved oral fluency over sustained training periods.

Industrial applications highlight NVIDIA Omniverse’s [171] use of GANs for synthetic factory fault datasets, enabling predictive maintenance systems that reduce equipment downtime by 18% and annual maintenance costs by $2.1 million in Siemens2 pilot facilities. The entertainment sector showcases Epic Games’ MetaHuman tool, powered by Transformers, which dramatically cut character design costs while attracting millions of users to virtual concerts. Retail innovations include Alibaba’s GAN-driven virtual fitting rooms Outfit Anyone [172], decreasing return rates by 25% and boosting conversion rates by 18% on e-commerce platforms.

Sociologically, Gucci’s AIGC-generated digital fashion items [173] achieved significant commercial success with a far lower carbon footprint compared to physical counterparts, underscoring sustainability benefits. These cases collectively illustrate AIGC’s role in enhancing efficiency, scalability, and user engagement within the Metaverse, while addressing critical challenges such as cost reduction, environmental impact, and personalized experiences.

### Challenges in AIGC technology development

The development and integration of AIGC technology within the Metaverse presents a range of complex challenges, both technical and ethical, that need to be carefully navigated. One of the most significant obstacles is the solution space complexity, which pertains to the difficulty of identifying and generating specific subspaces tailored for tasks like face or body generation. As the Metaverse demands diverse and highly detailed virtual content, ensuring macroscopic consistency is a major challenge, particularly in video generation. Limited fields of view in video rendering can disrupt underlying structures, resulting in a lack of continuity in long-term motion or structural consistency. On a microscopic level, maintaining clarity in generated content, especially in short video formats, is another issue, as averaging feasible solutions often leads to poor resolution and blurring. To tackle these hurdles, improvements in subspace optimization, such as the use of deep learning embeddings, meta-learning, and swarm intelligence, are essential. To address microscopic clarity issues, techniques like super-resolution, GANs, and blur removal algorithms can be employed to enhance the sharpness and detail of generated content. Additionally, the current entry into the Metaverse largely relies on highly immersive XR (extended reality) [174] (VR/AR/MR [mixed reality]) devices. However, the existing VR technologies struggle to miniaturize, make these devices portable, and reduce their costs, hindering users from accessing the Metaverse anytime and anywhere. At the same time, prolonged use of XR devices can cause discomfort.

Additionally, the integration of AIGC into the Metaverse introduces significant challenges related to energy consumption and sustainability, which merit closer attention. Training large-scale AIGC models, particularly for video generation and cross-modal integration, requires substantial computational resources, resulting in high energy costs and a considerable environmental footprint. The shift toward sustainability in AI development [175,176] is imperative, with potential solutions including the adoption of energy-efficient model architectures, such as sparsity-based neural networks, and the use of green cloud computing powered by renewable energy sources. Innovations in training optimization, such as model distillation and federated learning, can further reduce energy demands.

Another critical aspect is the balance between user agency and control in AIGC-driven systems. While AIGC empowers creators with automated tools [177–179], users may feel detached from the creative process if systems lack interactivity or customization options. User-in-the-loop frameworks, where individuals can iteratively refine generated outputs, are key to addressing this issue. For example, real-time tools that allow users to adjust parameters such as style, tone, or resolution would enhance personalization and creative autonomy. Additionally, intuitive interfaces for AIGC systems could democratize content creation, enabling non-experts to actively participate in the design of virtual environments, characters, and experiences, further enriching the Metaverse.

Finally, interoperability and real-time generation are technical challenges that must be overcome to realize the full potential of the Metaverse. As a decentralized and interconnected ecosystem, the Metaverse requires that AIGC-generated content be compatible across various platforms and devices. Developing universal standards for asset creation and exchange will be essential to ensure seamless integration. Real-time content generation, a cornerstone of interactive and immersive experiences such as live events or adaptive virtual assistants, demands low latency and high computational efficiency. Solutions such as edge computing [180–182], distributed architectures [183], and lightweight generative models [184] are critical to addressing these challenges. These issues still require further in-depth research.

Alongside these technical challenges, there are significant concerns around the ethical implications of AIGC’s rapid content generation. One of the key issues is related to copyright and intellectual property (IP) in the Metaverse [185], as AIGC can produce high volumes of content quickly [186], raising questions about ownership and originality. The realism of AIGC-generated content also increases the risk of misinformation, as it becomes easier to create and disseminate fake news, deepfakes, and other manipulated media. Protecting IP and preventing the spread of false information require robust solutions, such as embedding digital watermarks in AIGC-generated content for copyright verification and authenticity. Moreover, developing advanced AIGC detection systems to identify and flag fabricated content is crucial to maintaining trust in digital ecosystems. Legal frameworks are necessary to ensure fair distribution of benefits and safeguard creators’ rights [187–189], while regulatory transparency will foster a more secure digital environment by requiring AIGC systems to disclose the methods and data sources used in content generation.

Some organizational platforms are also actively promoting AIGC copyright protection norms, such as the World Intellectual Property Organization [190], which is researching the copyright legal framework for AI-generated content to provide transnational guidance; YouTube [191] and Getty Images, which use embedded watermarking and content identification technologies to protect the rights and interests of the originators; and OpenSea and Rarible, which protect the rights and interests of the original creators by casting AIGC generated digital artworks cast as NFTs, binding ownership information, and ensuring their traceability, and have realized digital transactions and management of art copyrights [192]. However, the originality and authorship of AI-generated content is recognized, and the copyright laws of many countries require works to have the participation of human creators, while the identity of the creators of AI-generated content has not been clarified, and this aspect of the law is relatively ambiguous [178]. Moreover, AIGC-generated content may use other people’s copyrighted material without authorization and the number of contents is large [193], which leads to the risk of copyright infringement and the protection of copyright registration also becomes complicated. Fortunately, a variety of mitigation strategies have been proposed. Technologically, numerous deepfake detection methods have been developed, ranging from CNNs and attention-based models to frequency-domain analysis and Transformer-based frameworks. These methods aim to identify subtle artifacts or inconsistencies in generated content. In parallel, legislative actions are being implemented in several countries, such as the DEEPFAKES Accountability Act in the United States, which mandates clear labeling and imposes penalties for malicious use. Additionally, initiatives like the Content Authenticity Initiative (CAI) seek to establish content provenance by embedding metadata and leveraging blockchain for verification. Public education campaigns are also crucial in enhancing media literacy, helping individuals critically assess the authenticity of digital media. Lastly, social media platforms have introduced stricter moderation policies to detect and remove deepfake content, thereby reducing its spread and impact.

In the face of the above challenges, the AIGC copyright management mechanism in the meta-universe can generate a unique digital fingerprint, i.e., assign a unique hash value to each AIGC content through smart contract and blockchain technology, and record the time of its creation, creator, and right of use information on the blockchain to ensure traceability, so as to guarantee the copyright ownership of user-generated content in the meta-universe and adapt to the complex ecology of multi-party participation [194]. It realizes the automation and efficiency of copyright management and avoids the tedious process of traditional copyright transactions. It can also compare content features based on deep learning models and match them with the existing content in the database, through which potential copyright conflicts or infringements can be detected. These issues still require further in-depth research.

Looking to the future, AIGC technology has the potential to markedly shape the development of the Metaverse, particularly in content creation, scalability, and interactivity. As AIGC systems evolve, they will foster new levels of creativity and collaboration between humans and AI. AIGC will empower creators, developers, and users to produce multi-modal content, including text, images, video, and even interactive experiences, allowing for highly personalized and engaging content. This collaboration will lead to an explosion of innovation and diverse content that can be seamlessly integrated into the Metaverse. The scalability of AIGC models will allow for rapid, cost-effective content production, enabling the Metaverse to expand rapidly and offer increasingly dynamic virtual environments. With the integration of cloud computing, the computational resources required for AIGC models will be markedly optimized, reducing training and inference times while also lowering costs. This cloud-based approach will support the large-scale deployment of AIGC technologies, ensuring that immersive experiences can be delivered to users with minimal delay.

Additionally, cross-modal integration will also drive innovation within the Metaverse, particularly in areas like virtual education, healthcare, and entertainment. By combining multiple forms of data—text, images, audio, and video—AIGC models will be able to create more cohesive and interactive experiences, enhancing user engagement. Furthermore, future AIGC systems will be designed with enhanced intelligence and adaptability, capable of independent learning and decision-making. This autonomy will ensure that the content generated remains accurate, relevant, and aligned with user needs, providing a richer and more personalized experience. As these systems become more autonomous, they will also integrate privacy-preserving technologies like federated learning and encryption, ensuring that user data remains secure.

Ultimately, AIGC will play a central role in transforming industries by enabling cost-effective content creation, accelerating innovation, and driving economic growth. Fields such as cultural tourism, education, healthcare, and industrial manufacturing will all benefit from the ability to generate complex, immersive content quickly and efficiently. In the Metaverse, AIGC will serve as a powerful tool for cross-industry collaboration, offering new opportunities for businesses and users to interact, create, and innovate. By overcoming the challenges outlined above and leveraging the full potential of emerging technologies, AIGC will redefine how we experience and interact with digital worlds. As AIGC continues to evolve, its integration into the Metaverse will be essential in creating vibrant, dynamic, and innovative digital environments.

## Discussion

Building on a summary of the current strengths and limitations of AIGC applications in the Metaverse, we propose the development of a more comprehensive and detailed evaluation framework. This framework should not only address the complexities of virtual environments but also thoroughly assess the quality of AIGC-generated content, the level of immersive user experiences, and the innovative potential of future model-centric advancements. AIGC holds transformative potential for the Metaverse, enabling the synthesis of complex and realistic data through computational methods to address challenges in Metaverse content creation. This study begins by examining the core neural network architectures of GAI, analyzing the development of deep learning technologies behind AIGC, and exploring the relationship between the Metaverse and AIGC. Additionally, the study highlights the application prospects and challenges of AIGC in fields such as healthcare, education, and sociology. Although AIGC can markedly accelerate the development of the Metaverse, its technology must be better aligned with practical development needs to achieve more effective applications. This research aims to provide a foundational knowledge framework for academic researchers and industry practitioners, thereby advancing the evaluation methods and application technologies of AIGC in the Metaverse to new heights.

### Where is the Metaverse today?

Arguably, despite its immense promise, the Metaverse has yet to live up to the initial hype. One of the primary challenges lies in the current technological and infrastructural limitations that still hinder the seamless, immersive experiences promised by early proponents of the technology. One of the central technologies of an immersive Metaverse, VR headsets, face particular challenges with high costs and user comfort issues—many people report motion sickness, eye strain, and general discomfort during extended sessions, and while the Metaverse concept extends beyond VR, this remains a particular concern, despite potential accessibility through PCs, mobile devices, and AR; even these more accessible entry points have not gained significant traction [195].The Metaverse’s development has likely also been slowed down by insufficient content creation and consumer uptake, including in the areas described above, leaving virtual environments feeling empty, repetitive, and underwhelming compared to the dynamic worlds envisioned. The absence of interoperability between platforms has further fragmented the user experience, preventing the creation of a unified digital ecosystem. Additionally, the emergence of competing technologies has further complicated the Metaverse’s trajectory. The explosive rise of GAI has captured public imagination and diverted both attention and investment that might otherwise have gone to entirely to metaverse development. This shift in focus has been particularly impactful as it coincides with growing societal skepticism about the Metaverse, stemming from concerns about data privacy, cybersecurity, and ethical implications. However, while mass adoption remains uncertain, the Metaverse is still finding success in specific niches. Virtual training platforms are being adopted by industries ranging from healthcare to manufacturing, offering safe, cost-effective environments for skill development. Educational institutions are exploring virtual campuses and immersive learning experiences, while gaming platforms like Roblox and Fortnite continue to build engaged communities around shared virtual experiences. The concept of the Metaverse itself continues to evolve. The initial hype may have been premature, setting unrealistic expectations for immediate transformation across all sectors. As technology advances and expectations become more grounded, we believe that the Metaverse might gradually find its footing. This could perhaps involve a more distributed and interoperable network of virtual spaces, rather than the single, unified virtual world initially envisioned by early evangelists. In all likelihood, people simply prefer simpler, more accessible forms of digital interaction for daily use, indicating that the Metaverse’s eventual role might be complementary to, rather than replacing, existing digital experiences. As the Metaverse navigates these challenges and finds its footing in specific niches, it is crucial to address the ethical, legal, and social implications (ELSI) of its development. The obstacles to widespread adoption extend beyond technological and economic factors to include deeper societal concerns that influence how the Metaverse is perceived and utilized. Issues such as data privacy, cybersecurity, and equitable access must be carefully examined to ensure that this evolving digital ecosystem can be both trusted and inclusive. Moreover, questions of governance, identity, and the potential for social stratification within virtual spaces continue to highlight the need for a comprehensive framework to guide the responsible growth of the Metaverse. Exploring these ELSI aspects will help illuminate the broader challenges and opportunities inherent in shaping the future of this digital frontier.

### ELSI of GAI in the Metaverse

As the Metaverse, in whatever iteration it is currently or eventually will be in, integrates GAI (AIGC) to build immersive virtual ecosystems, its rapid development presents a myriad of ethical, legal, and social challenges. Addressing these implications is critical to ensure the Metaverse remains equitable, inclusive, and responsibly governed. This section of the discussion explores these considerations across various dimensions, with a focus on healthcare, education, and broader societal impacts.

#### Ethical considerations

Generative AI systems in the Metaverse must navigate issues of bias and fairness. AIGC relies on vast datasets, which, if poorly curated, can reinforce existing societal biases perpetuating stereotypes and marginalizing underrepresented groups. Developing diverse and representative training datasets and incorporating fairness algorithms are crucial steps toward addressing these issues.

Another major ethical concern is privacy. The immersive nature of the Metaverse involves extensive collection of personal and behavioral data both prior to use and in real time during use. This raises significant risks of surveillance and misuse by governments, corporations, and perhaps even malicious entities. Privacy-preserving technologies, such as federated learning and encryption, can mitigate these risks by decentralizing data storage and limiting unnecessary data collection.

The Metaverse’s immersive experiences also raise concerns about dependency and addiction. Prolonged engagement with virtual environments can lead to social withdrawal, disrupted real-world relationships, and other mental health challenges. Developers should integrate features to promote healthy digital habits, such as usage limits, well-being reminders, and tools for maintaining a balanced engagement between virtual and real-world activities [196].

#### Legal challenges

The legal landscape of the Metaverse is still evolving, with significant gaps in addressing IP and accountability issues. Ownership of AIGC-generated assets—such as virtual environments, avatars, or digital art—is often ambiguous, and challenging traditional IP frameworks as AI can neither invent nor author basic fundamental requirements for obtaining patents and copyrights, respectively [197,198]. Policymakers must establish clear guidelines to determine ownership of places and things generated via AIGC in the metaverse, ensuring creators’ rights are protected while fostering innovation. Accountability is another pressing issue. If harm arises from biased or harmful AI-generated content, defining responsibility becomes complex. Developers, platform operators, and AI designers must work together to create transparent systems for easily assigning liability. Additionally, the global nature of the Metaverse demands international cooperation to establish consistent regulations across jurisdictions for these and other legal concerns.

#### Health implications

In healthcare in particular, the Metaverse, augmented by AIGC, offers transformative opportunities, but it also offers significant risks. Virtual healthcare environments powered by AIGC can enhance medical training through realistic simulations, improve telemedicine experiences, and generate synthetic datasets for research. However, overreliance on AI-generated content for diagnoses or treatment recommendations poses risks of inaccuracies and malpractice, particularly when algorithms lack sufficient training or context. Ethical safeguards, such as rigorous testing of AIGC systems and transparency in decision-making, are critical to ensure patient safety. Privacy concerns are also particularly acute in healthcare, as sensitive medical data could be exploited if security measures are insufficient. Blockchain-based systems and federated learning approaches can address these concerns by providing secure, decentralized solutions.

#### Education implications

As described above, the Metaverse also holds vast potential in education, where AIGC can create immersive, interactive learning experiences. Virtual classrooms, customizable simulations, and experiential learning tools can help students engage with complex subjects more effectively. However, disparities in access to such technologies risk exacerbating the digital divide [199]. Policymakers and educators must prioritize affordable solutions and ensure that virtual learning environments are inclusive. Additionally, overreliance on AIGC-driven education tools may lead to reduced critical thinking skills if students depend solely on automated systems. To address this, educators should blend AIGC with traditional teaching methods to foster a balanced, well-rounded educational experience.

### Proposed strategies for responsible development

Addressing these challenges requires a multifaceted approach. Ethical AI design must prioritize fairness, explainability, and accountability, with regular audits to ensure AIGC systems operate responsibly. Privacy-preserving technologies, such as encryption and decentralized storage, should be integral to Metaverse platforms. International cooperation is essential to create legal frameworks that address cross-border complexities, particularly around IP and liability. Finally, public education campaigns can raise awareness about the ethical and social dimensions of the Metaverse, empowering users to engage responsibly with this evolving digital frontier.

Ultimately, by embedding ethical, legal, and social considerations into the fabric of Metaverse development, i.e., ethics by design, stakeholders can create a digital ecosystem that is not only innovative and immersive but also inclusive, equitable, and sustainable. These efforts will ensure the Metaverse evolves into a trusted and empowering space for all users.

## Data Availability

Data will be made available on request.
